# Reconstructed Monolithic RuNi Heterostructure Enables Hydrogen Production from Alkaline Seawater at Industrial Current Density

**DOI:** 10.1002/advs.202510916

**Published:** 2025-10-06

**Authors:** Hao Luo, Tao Zhou, Ruiqin Xia, Zhengxiao Guo

**Affiliations:** ^1^ Department of Chemistry The University of Hong Kong Hong Kong SAR P. R. China

**Keywords:** alkaline seawater electrolysis, hydrogen evolution reaction, monolithic electrode, RuNiOx composite, surface reconstruction

## Abstract

Industrial‐scale hydrogen production requires electrocatalysts capable of simultaneously delivering high activity, long‐term durability, and seawater compatibility. In this work, a monolithic RuNiO_x_ catalyst directly grown on Ni foam is reported, which evolves into the Ru/NiO heterostructure during operation. The reconstructed electrocatalyst presents excellent catalytic performance with a mass activity of 0.47 A mg^−1^_Ru_ at an overpotential of 50 mV, twice that of the benchmarked Pt/C (0.21 A mg^−1^_Pt_) and remarkable durability of over 350 h at 1 A cm^−2^ without noticeable degradation. Even in harsh alkaline seawater, the electrode maintains stable operation for 100 h at both 100 and 500 mA cm^−2^, and functions reliably at 65 °C under industrial conditions. In situ spectroscopic and computational results reveal that RuO_2_ is dynamically reduced to metallic Ru while NiO remains stable, thereby forming robust Ru/NiO interfaces as the real active site. This study demonstrates an effective strategy for designing high‐performance electrodes suitable for industrial‐scale seawater electrolysis.

## Graphical abstract

In situ grown monolithic RuNiO_x_ on nickel foam is reconstructed under cathodic conditions to yield a robust Ru/NiO heterointerface. This evolution optimized the surface hydrophilic‐aerophobic properties and tuned the adsorption affinity for key intermediates, thus delivering exceptional activity and durability for the hydrogen evolution reaction in alkaline seawater at industrial current densities.

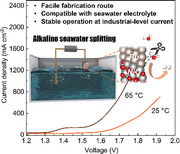

## 1 Introduction

Hydrogen is an ideal alternative to fossil fuels and has gained considerable attention as a sustainable clean energy carrier due to its high energy density, abundant resources in water, and net‐zero carbon emission.^[^ ^1^, ^2^, ^3^ ^]^ Alkaline electrochemical water splitting is regarded as one of the most promising approaches to generate hydrogen, yet progress is hindered by the high cost of platinum, known as the best electrocatalyst for cathodic hydrogen evolution reaction (HER).^[^ ^4^, ^5^ ^]^ Considerable efforts have been devoted to cost‐effective electrocatalysts to replace Pt, and indeed substantial success has been achieved, at least on the laboratory scale.^[^ ^6^, ^7^, ^8^, ^9^, ^10^, ^11^ ^]^ To step up further toward practical applications, high‐performance catalysts are required to match well with industrial conditions. Specifically, HER electrocatalysts should possess at least the following characteristics:^[^ ^12^, ^13^, ^14^, ^15^, ^16^ ^]^ 1) scalable synthesis with inexpensive raw materials; 2) stability under harsh operational conditions, such as large current (>0.5 A) and high temperature; and 3) highly efficient performance and compatibility with seawater‐derived electrolytes.

However, the above criteria are rarely satisfied together by one type of electrocatalyst alone. For example, single‐atom catalysts can maximize the catalytic activity of a single catalytic site, yet their preparation usually involves multi‐steps and rigorous routines, not easy to scale up.^[^ ^17^, ^18^, ^19^, ^20^ ^]^ Moreover, the existing criteria for lab‐scale assessment of HER catalysts seem to focus on the overpotential to drive the current density to 10 or 50 mA cm^−2^.^[^ ^21^ ^]^ Once the current density goes up to the ampere level, rapidly generated bubbles can readily accumulate on the catalyst surface and thus shield the reaction sites.^[^ ^22^ ^]^ Moreover, potential mechanical issues also arise under a large current density, such as nanostructural agglomeration and detachment of powder‐type catalysts from the electrode surface.^[^ ^23^ ^]^ Furthermore, lab‐based electrolytes for alkaline water electrolysis are usually formulated from high‐purity water with nonreactive metal ions for ionic conductivity. From the perspective of techno‐economics, solar‐driven seawater electrolysis is more attractive, as the electrolyte comes directly from seawater.^[^ ^24^, ^25^, ^26^, ^27^ ^]^ As natural seawater contains metal cations, once involved in a large current density, the local pH near the electrode surface increases considerably, leading to the formation of metal hydroxide precipitates on the electrode surface.^[^ ^2^, ^28^ ^]^ Although the prevalent pretreatment of seawater by hydroxide to eliminate metal cations, the complexity of seawater still challenges the use of existing electrocatalysts with regards to both activity and durability.^[^ ^29^ ^]^

Combining theory and experiment to reveal catalysis mechanism on a solid surface is significantly helpful to screen highly‐efficient HER electrocatalysts.^[^ ^8^ ^]^ An ideal electrocatalyst for alkaline HER should efficiently disassociate water molecules and facilitate the molecular hydrogen formation simultaneously.^[^ ^5^, ^30^ ^]^ Ruthenium (Ru)‐derived electrode materials are widely reported for alkaline water splitting as their unique physicochemical properties are close to Pt, yet at a lower price and better hydrolytic dissociation ability.^[^ ^31^, ^32^, ^33^, ^34^, ^35^ ^]^ Given the fact that Ru‐H bond strength is too strong to release adsorbed hydrogen, H^*^, modulating the Ru‐H interaction is a common practice to optimize Ru‐derived electrode for alkaline HER.^[^ ^31^, ^33^, ^36^ ^]^ For example, P, Mo dual‐doped Ru nanoparticles enveloped in P‐doped porous carbon (P, Mo‐Ru@PC) showed an extremely low overpotential of 21 mV to produce a current of 10 mA cm^−2^ and a Tafel slope of 21.7 mV dec^−1^ in 1 m KOH.^[^ ^37^ ^]^ DFT calculations indicated that the electron transferred from Ru to Mo and P modulated the Gibbs free energy of H adsorption (Δ*G*_H*_) to change from −0.45 eV on Ru@C to −0.28 eV on P, Mo‐Ru@PC. Furthermore, Sr_2_RuO_4_ bulk single crystals achieved a large current density of 1 A cm^−2^, at an overpotential of 278 mV in 1 m KOH,^[^ ^38^ ^]^ and the superior performance was attributed to in situ evolved Ru cluster‐Sr_2_RuO_4_ structure, which adjusted the Δ*G*_H*_ from −0.45 eV on bulk Ru to −0.12 eV on Ru cluster‐Sr_2_RuO_4_. Wu et al. anchored Ru nanograins on NiMoO(P)_4_ nanosheet array supported on Ni foam,^[^ ^39^ ^]^ and the electrode exhibited an extremely low overpotential of 24 mV at a current density of 10 mA cm^−2^ with a small Tafel slope of 34 mV dec^−1^. The overall alkaline seawater electrolysis was also assessed with the electrode, requiring a cell voltage of 1.78 V to reach 100 mA cm^−2^ in 1 m KOH seawater. These reports indeed advance the understanding of Ru‐based electrocatalysts for alkaline HER. While the systematic investigation of Ru‐based electrocatalysts for alkaline HER and seawater electrolysis is still sparse, especially with an emphasis on structural evolution and structure‐performance relationships.

Here, we reported a facile strategy for constructing monolithic ruthenium‐nickel oxide (RuNiO_x_) nanocomposites grown on nickel foam (NF) as a high‐performance electrocatalyst for alkaline seawater splitting. The electrode exhibited exceptional HER activity with an ultralow overpotential of 204 mV at 1000 mA cm^−2^ and a small Tafel slope of 21.3 mV dec^−1^. It demonstrated outstanding durability, maintaining stable operation for over 350 h at a high current density of 1000 mA cm^−2^ with minimal potential decay. Structural characterization and in situ spectroscopy revealed that under cathodic conditions, RuNiO_x_ underwent dynamic reconstruction, forming a robust metallic Ru/NiO interface that served as the highly active site for HER. Furthermore, the electrode showed excellent compatibility with alkaline seawater, enabling continuous operation at 500 mA cm^−2^ for 100 h and stable performance at an industrial temperature of 65 °C.

## 2 Results and Discussion

### 2.1 Characterizations

To fabricate a catalytic electrode capable of operating at high current densities for alkaline water electrolysis, several critical factors must be considered. First, the active catalytic components must exhibit high efficiency and robust stability for the HER under alkaline conditions. Second, the electrode structure should resist delamination caused by rapid gas bubble generation and maintain structural integrity under harsh operating conditions. Third, the catalyst must maintain firm contact with the current collector to ensure efficient electron transport under high‐current operation. Fourth, the electrode should possess appropriate wettability to ensure intimate electrolyte‐catalyst contact and promote efficient bubble release.

**Figure 1:**
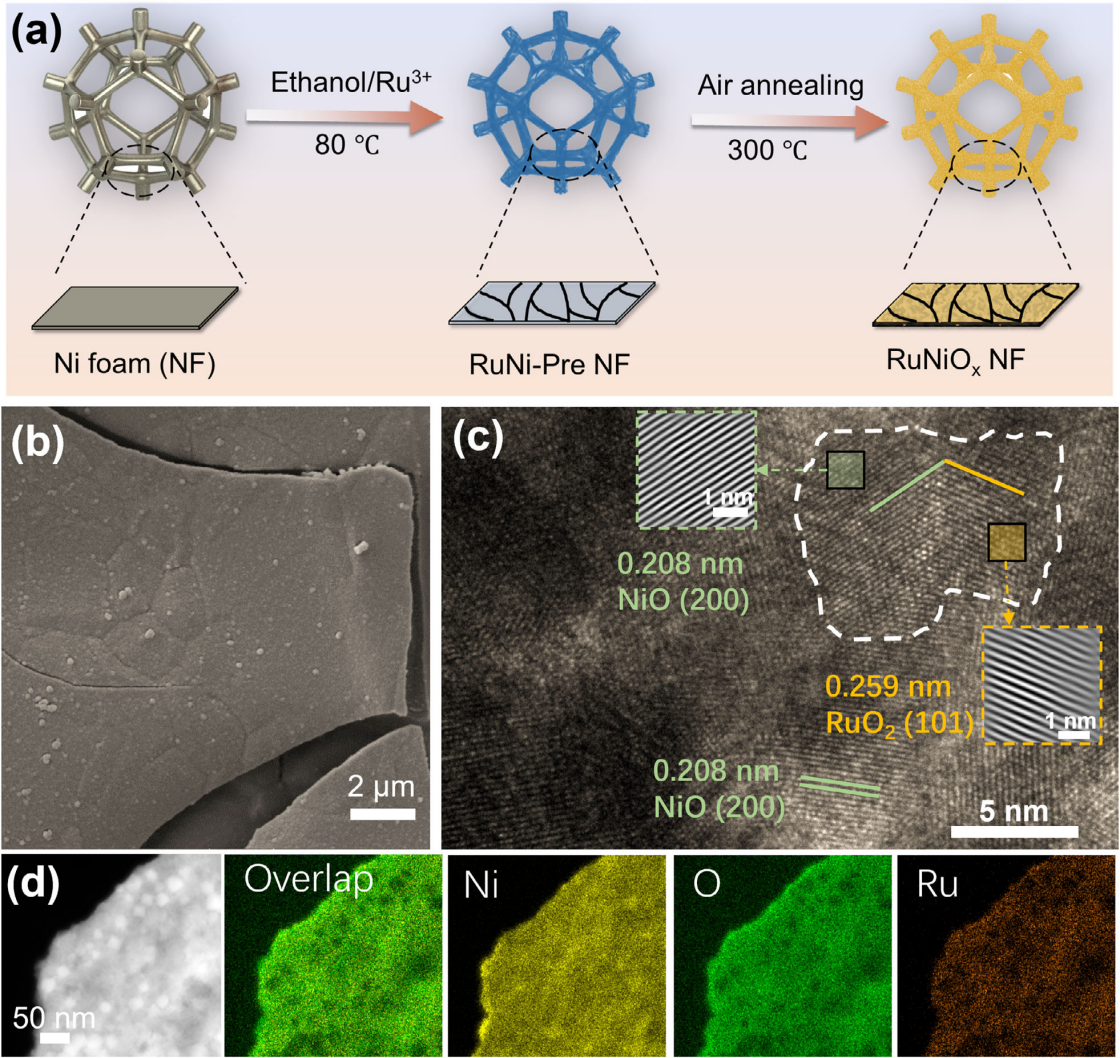
a) Schematic illustration of the fabrication process of the RuNiO_x_. b) SEM of RuNiO_x_. c) High‐resolution TEM image, inset: inverse FFT images. d) Elemental mapping images.

With the above in mind, we proposed to fabricate a monolithic RuNiO_x_, generated in situ from a Ni‐foam electrode. The synthesis of RuNiO_x_ involved two facile steps (**Figure** **1**a). First, a piece of nickel foam (NF) was used as the conductive substrate and nickel source, and ruthenium chloride hydrate as the ruthenium source. Here, the Ru^3^⁺ ions chemically etched the NF substrate, releasing Ni^2^⁺ ions into the solution for the subsequent co‐precipitation. Following a reaction duration of 8 h at 80 °C, this process resulted in a uniform dark black layer of RuNi precursor (RuNi‐Pre) thoroughly penetrating the micro‐structured surface of the NF, creating a mechanical interlock and ensuring robust physical adhesion (Figure S1, Supporting Information). Second, the electrode was subjected to thermal treatment at 300 °C in air to obtain stable metal oxides, hereafter denoted as RuNiO_x_. The annealing process not only dehydrated the above precursor to form oxides but also facilitated the formation of strong covalent M─O bonds at the interface, thereby achieving robust chemical integration between the catalyst layer and the NF substrate. The effects of thermal treatment on morphology were examined by SEM (Scanning Electron Microscopy) and XRD (X‐Ray Diffraction). Compared with surface of pristine NF (Figure S2, Supporting Information), macroscopic fragment assembled by dense nanoparticles appeared on the topmost surface (Figure 1b). While the morphology of electrodes without adding Ru sources during the synthesis displayed numerous nanoflakes (Figure S3, Supporting Information). It can be visualized from element mapping that both Ru and Ni in the RuNiO_x_ were homogeneously distributed throughout the skeleton (Figure S4, Supporting Information). XRD patterns indicated that the as‐synthesized RuNi precursor exhibited an amorphous feature (Figure S5, Supporting Information). After annealing, several new peaks emerged. Peaks at 37.3, 43.2, and 62.9° can be assigned to the (111), (200), and (220) crystalline planes of cubic NiO (JCPDS No. 04–0835), respectively. While peaks at 28.2, 35.2, and 54.3° corresponded to (110), (101), and (211) of tetragonal RuO_2_ (JCPDS No. 88–0322), respectively. These results confirmed the co‐existence of NiO and RuO_2_ on the annealed electrode. The XRD results were consistent with the proposed reaction mechanism, where RuCl_3_ and Ni atoms reacted to form a RuNi precursor (RuNi‐Pre), which was subsequently converted to NiO‐RuO_2_ upon thermal annealing.

Transmission electron microscopy (TEM) was employed to provide further details of the microstructure (Figure S6, Supporting Information). The as‐prepared RuNi‐Pre showed poor crystallinity, as confirmed by the high‐resolution TEM and selected area electron diffraction (SAED) (Figure S6a,b, Supporting Information). While clear lattice fringes were observed on the sample after annealing (Figure 1c). The high‐resolution TEM image provided clear evidence for the formation of a well‐defined NiO/RuO_2_ heterojunction. The lattice spacings of 0.208 and 0.259 nm were assigned to the (200) plane of NiO and the (101) plane of RuO_2_, respectively. The coherent interfacial contact between these two distinct crystalline phases created favorable pathways for rapid charge transfer and induced electronic modulation across the heterogeneous interface, which was expected to contribute to the enhanced electrocatalytic activity.^[^ ^40^, ^41^, ^42^, ^43^ ^]^ The coexistence of both nickel oxide and ruthenium oxide phases was further confirmed by SAED analysis (Figure S6d,f, Supporting Information), consistent with the HRTEM observations. TEM images also revealed that irregular nanograins with the size of 25 nm were uniformly distributed on RuNiO_x_, while electrodes fabricated at a higher anneal temperature of 500 °C (RuNiO_x_‐500) exhibited serious agglomeration of particles with a size > 50 nm (Figure S6c,e, Supporting Information). Besides, the corresponding energy dispersive X‐ray (EDX) mapping showed a homogenous distribution of Ni, Ru, and O elements on RuNiO_x_ (Figure 1d), as also confirmed by the X‐ray photoelectron spectroscopy (XPS) (Figure S7, Supporting Information). The mass loading of ruthenium on the electrode was quantitatively determined through inductively coupled plasma optical emission spectrometry (ICP‐OES), revealing a relatively low level of 0.245 mg_R__u_ cm^−2^.″

### 2.2 HER Performance

**Figure 2:**
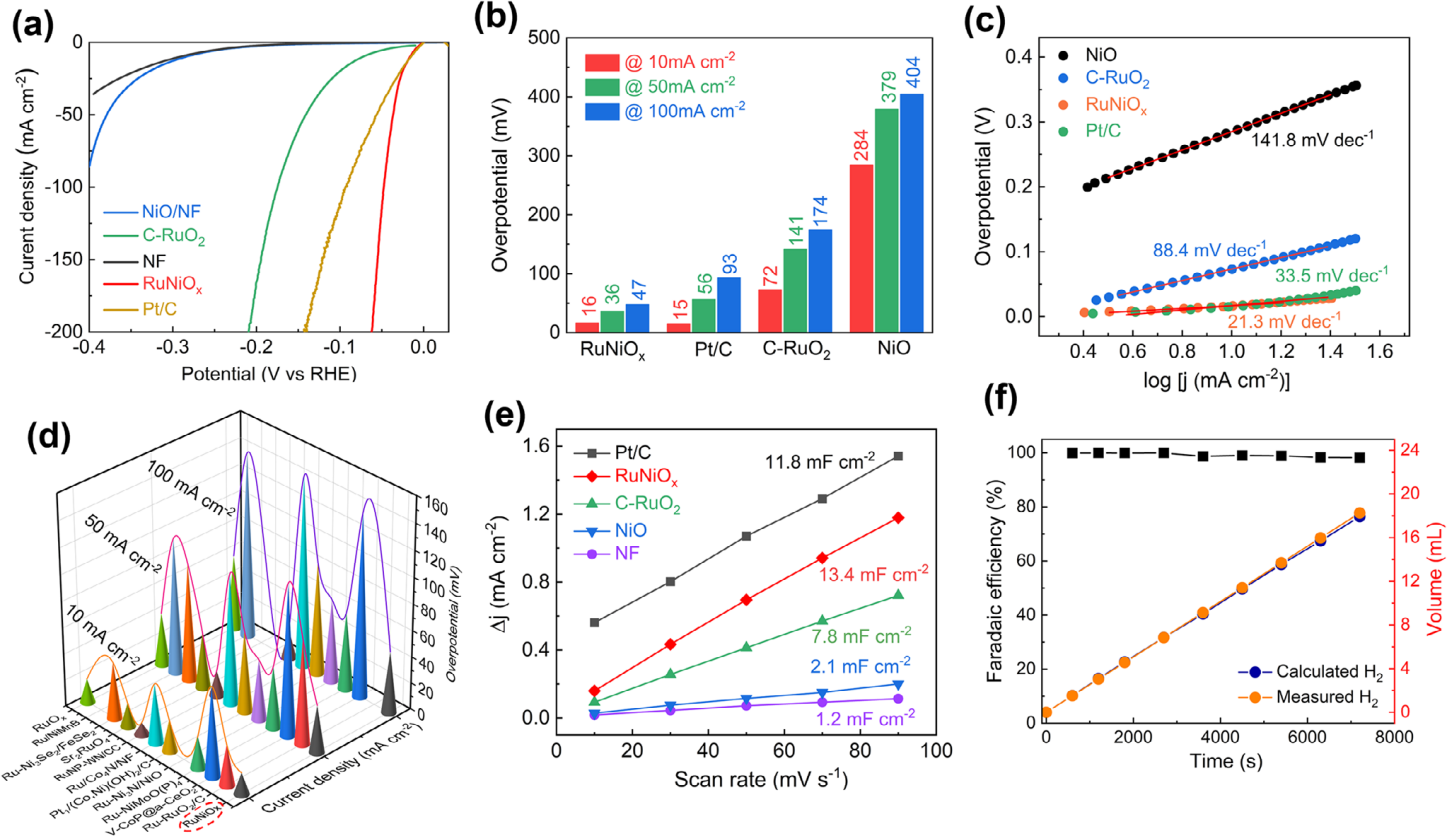
a) Polarization curves of NF, NiO/NF, C‐RuO_2_, RuNiO_x_, and Pt/C in 1 M KOH. b) Comparison of overpotentials at different current densities. c) Corresponding Tafel slope. d) Comparison of RuNiO_x_ with recent state‐of‐the‐art electrodes. e) Current versus scan rate in the non‐faradic region. f) Faradaic efficiency measurement of the RuNiO_x_ electrode for HER.

HER performance of RuNiO_x_ electrodes was examined using a three‐electrode system in 1.0 M KOH. Pristine NF, NiO, commercial RuO_2_ (C‐RuO_2_), RuNiO_x_‐500, and Pt/C were also evaluated for comparison (**Figure** **2**a; Figure S8, Supporting Information). As anticipated, the RuNiO_x_ electrode demonstrated the highest HER electrochemical activity, requiring overpotentials of 16, 36, and 47 mV to achieve current densities of 10, 50, and 100 mA cm^−2^, respectively. These values are lower than those of Pt/C, which required overpotentials of 15.0, 56, and 93 mV to achieve the same current densities (Figure 2b). At an overpotential of 50 mV, RuNiO_x_ exhibited a mass activity of 0.47 A mg^−1^_Ru_, which was 2.2 times that of the commercial Pt/C (0.21 A mg^−1^_Pt_). Considering that the price of Ru is ∼68% of Pt, these advantages further highlighted the potential of RuNiO_x_ as a high‐performance, cost‐effective alternative to Pt‐based electrocatalysts for the hydrogen evolution reaction (Supplementary Note). The RuNiO_x_ electrode demonstrated superior HER kinetics with a much smaller Tafel slope of only 21.3 mV dec^−1^, compared to its counterparts, such as Pt/C (33.5 mV dec^−1^), C‐RuO_2_ (88.4 mV dec^−1^), and NiO (141.8 mV dec^−1^) (Figure 2c). Such a low Tafel slope value on RuNiO_x_ suggested that it followed the Volmer‐Tafel mechanism in alkaline HER.^[^ ^44^, ^45^ ^]^ The catalytic performance of RuNiO_x_ also stood out from recent reported Ru‐based and Pt‐based catalysts (Figure 2d; Table S1, Supporting Information). We further compared the double‐layer capacitance to estimate the electrochemical surface area (Figure 2e; Figure S9, Supporting Information). The RuNiO_x_ catalyst exhibited a double‐layer capacitance (C_dl_) of 13.4 mF cm^−2^, over 10 times higher than the pristine Ni foam of 1.2 mF cm^−2^. The high C_dl_ value suggests that the RuNiO_x_ contains more accessible active sites for HER, allowing for more efficient charge transfer and faster reaction kinetics.^[^ ^46^ ^]^ In Figure 2f, the Faradaic Efficiency (FE) of the RuNiO_x_ was confirmed to be nearly 100%, manifesting that the observed current was indeed electrolytic hydrogen production.

**Figure 3:**
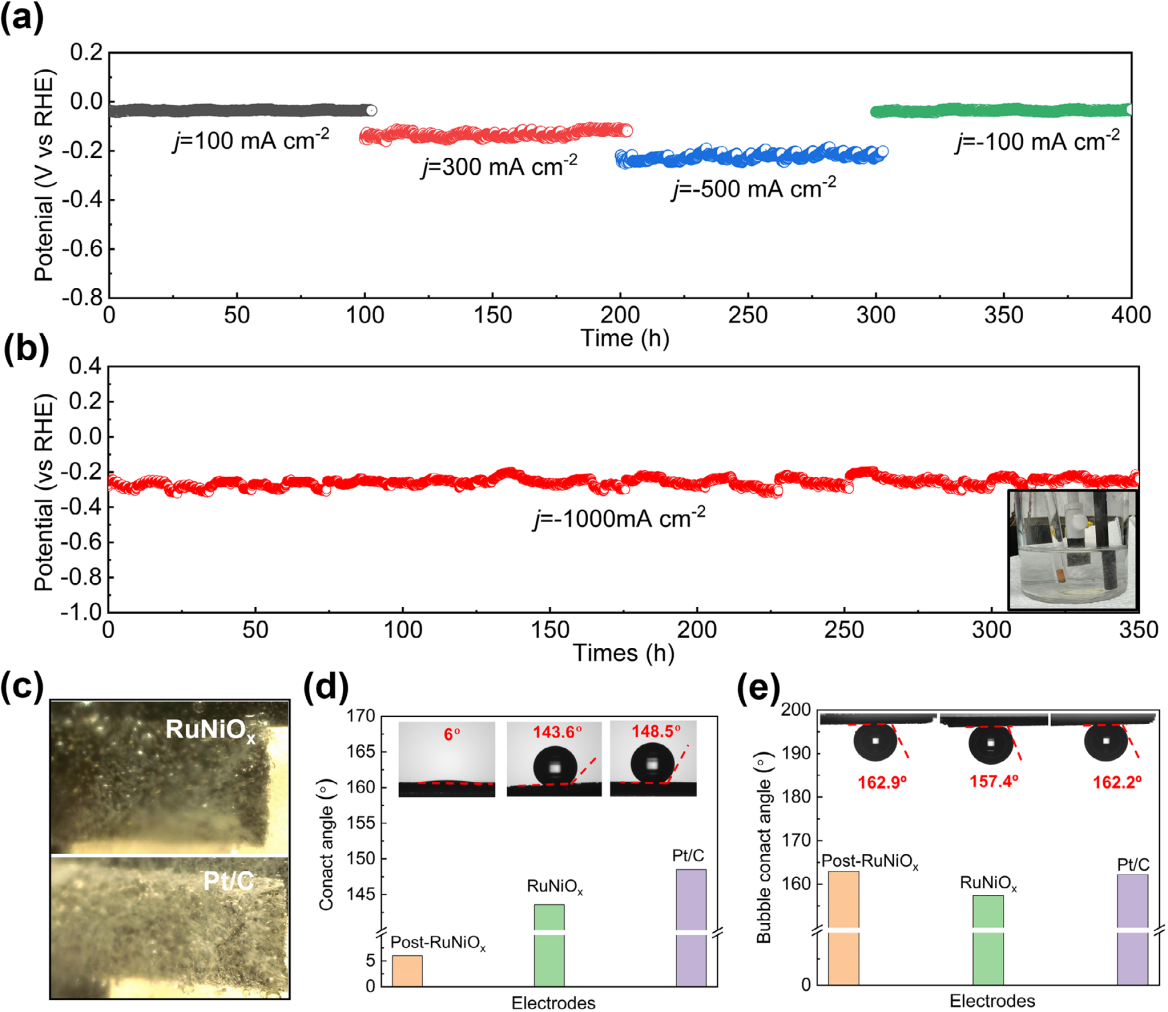
a) Long‐term stability tests at 100, 300, and 500 mA cm^−2^ in 1 m KOH electrolyte, respectively. b) Long‐term stability tests at 1000 mA cm^−2^, inset: photograph of electrolyzer vessel after stability test. (Note: the periodical fluctuation arose from the bubble release and accumulation). c) Photographs of Pt/C and Ru/NiO_x_ as electrode for HER in 1 m KOH electrolyte at 500 mA cm^−2^. d) Liquid contact angles, and e) Gas‐bubble contact angles of the post‐RuNiO_x_, RuNiO_x_, and Pt/C electrodes, respectively.

Durability of a catalyst is always a crucial factor in determining its overall efficacy, as it must maintain its performance under rigorous conditions and over extended periods of operation. To assess its stability, the RuNiO_x_ electrode was subjected to fixed current densities of 100, 300, and 500 mA cm^−2^, and then back to 100 mA cm^−2^ for 100 h per test. The result demonstrated stable operations without notable degradation at all the tested currents (**Figure** **3**a). Given its relevance to commercial and competitive applications, large current at the ampere level was also evaluated to assess its stability. As shown in Figure 3b, RuNiO_x_ maintained a nearly constant operating potential at 1000 mA cm^−2^ for over 350 h with no observable black mass in the bottom of electrolyzer vessel. For comparison, the stability of Pt/C supported on Ni foam was also evaluated. Pt/C electrode exhibited continuous performance degradation at 1000 mA cm^−2^ over 24 h and severe detachment of catalyst was observed, highlighting the poor mechanical stability of binder‐type electrodes under high current densities (Figure S10, Supporting Information). The outstanding stability of RuNiO_x_ at high current densities (>1000 mA cm^−2^) stemmed from its integrated design. The catalyst layer was intrinsically anchored within the Ni foam scaffold, creating a robust chemical and mechanical interlock that distributed stress and prevented localized peeling. Besides, one remarkable comparison of the structural differences between Pt/C and RuNiO_x_ electrodes during HER was captured in Figure 3c, showing rapid bubble release on the RuNiO_x_ surface, whereas lots of bubbles accumulated on the Pt/C surface. The properties were further quantified by the wettability test. Notably, we selected three electrodes for comparison, pristine RuNiO_x_, RuNiO_x_ after HER (post‐RuNiO_x_) and Pt/C. The pristine RuNiO_x_ showed a hydrophobic surface with a large contact angle (CA) of 143.6°, close to that of Pt/C (CA = 148.5°). In contrast, water droplets permeated swiftly upon contact with the post‐RuNiO_x_, underscoring its hydrophilic nature with a rather small CA of 6° for the electrolyte (Figure 3d). The change of hydrophilicity may indicate some dynamic evolution of the catalyst surface, serving as a telltale sign for us to do further postmortem analysis of the post‐RuNiO_x_, which we shall discuss in the next section. Moreover, the gas bubble CA was also measured and shown in Figure 3e. The contact angle of gas bubbles on post‐RuNiO_x_, RuNiO_x_, and Pt/C were 162.9°, 157.4°, and 162.2°, respectively. Thus, the remarkable durability was also enhanced by an evolved hydrophilic‐aerophobic surface. The hydrophilic property ensured efficient contact with active sites, while the aerophobic character facilitated ultrafast bubble release. This synergy significantly mitigated mechanical stress from bubble coalescence, which was the primary failure mechanism at high current densities, thereby preserving the stability of catalytic interface.^[^ ^22^, ^47^ ^]^

### 2.3 Structural Reconfiguration of the RuNiO_x_ Catalyst

**Figure 4:**
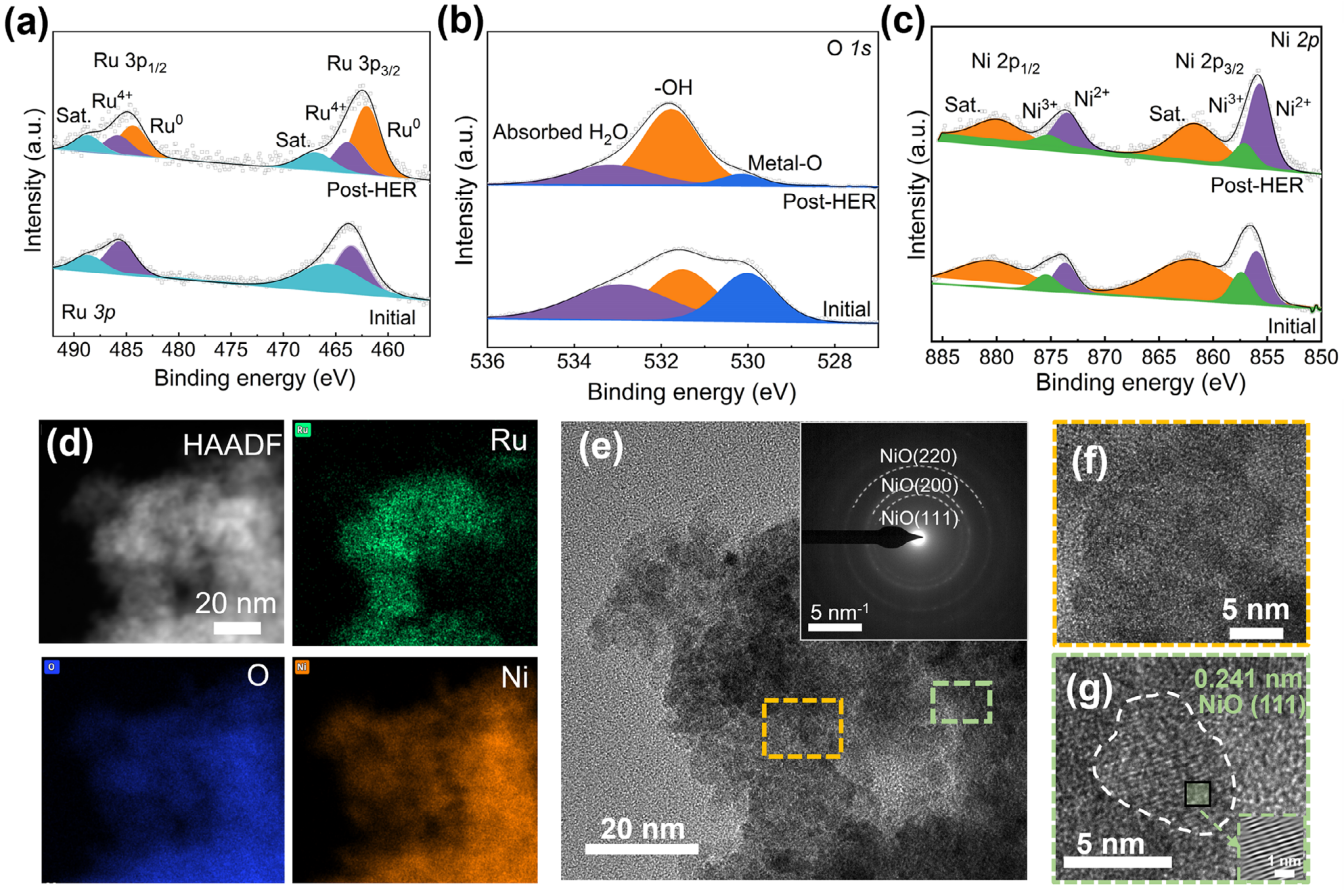
High‐resolution XPS spectra of initial and post‐HER electrode: a) Ru 3p spectra, b) O 1s, and c) Ni 2p. d) STEM mapping image of the post‐HER sample. e) The HRTEM image of the post‐HER sample, inset: SAED pattern of post‐HER sample. f) magnification picture of orange dash line box. g) magnification picture of green dash line box.

Reconfiguration of phase and composition is often observed in an electrocatalyst, especially when exposed to a reductive environment under a large current density.^[^ ^48^, ^49^, ^50^ ^]^ This reminded us to further detail real active sites of the RuNiO_x_ catalyst for HER. We performed post‐mortem analysis on an electrode tested at 1000 mA cm^−2^ for 350 h. SEM image revealed that the morphology remains neat after long‐term electrolysis while XRD pattern indicates only diffraction peaks corresponding to metallic Ni (from the Ni foam substrate) was detected, implying possible phase change of RuNiO_x_ during HER (Figures S11 and S12, Supporting Information). We first employed XPS to analyze changes in the surface chemistry after the stability test. The high‐resolution Ru spectra revealed that peaks at 463.5 and 485.6 eV corresponded to Ru 3p_3/2_ and 3p_1/2_, respectively, namely, Ru^4+^. For the post‐HER electrode, a pair of sharp peaks appeared at a lower binding energy region, at 462.1 and 484.4 eV, corresponding to metallic Ru^0^ (**Figure** **4**a).^[^ ^51^ ^]^ Quantitative analysis demonstrates a reconstruction of the Ru species under the reductive HER environment, with a Ru⁰/Ru⁴⁺ ratio reaching 2.23. Moreover, the O 1s spectra of RuNiO_x_ can be fitted into three peaks, namely metal‐oxygen (530.1 eV), hydroxyl (531.5 eV), and adsorbed oxygen (532.9 eV).^[^ ^52^ ^]^ In the post‐HER electrode, the reduction of RuO_2_ to Ru decreased the area ratio of metal–oxygen moieties, while the hydroxyl group coverage increased to 2.8 times that of pristine RuNiO_x_, thereby enhancing hydrophilicity (Figure 4b). This improved wettability facilitated the formation of a stable aqueous layer within the microstructure, which in turn enhanced aerophobicity by effectively repelling gas bubbles. Ni 2p spectra in RuNiO_x_ can be deconvoluted into spin‐orbit doublets including the binding energy at 855.8 (Ni 2p_3/2_) and 873.6 eV (Ni 2p_1/2_) assigned to Ni^2+^ and 857.4 (Ni 2p_3/2_) and 875.6 eV (Ni 2p_1/2_) to Ni^3+^.^[^ ^39^, ^53^ ^]^ While the higher energy couples are ascribed to satellite peaks (Sat.). For the post‐HER electrode, the Ni^2^⁺/Ni^3^⁺ ratio increased from 1.79 to 4.0 under the reductive condition, suggesting a reduction of Ni^3^⁺ to Ni^2^⁺ (Figure 4c). These concurrent valence changes of Ru and Ni indicate that under the reductive HER environment a clear reconstruction occurred on Ru─Ni sites with Ru⁴⁺ mostly reduced to Ru⁰ and Ni^3^⁺ stabilized as Ni^2^⁺. Furthermore, the reconstruction process and formation of the metallic Ru/NiO heterostructure were further visualized at the nanoscale. Scanning transmission electron microscopy (STEM) analysis was employed to characterize the post‐HER catalyst. High‐angle annular dark‐field (HAADF) imaging and the corresponding elemental mapping clearly revealed the spatially distinct but closely associated distributions of Ru and Ni elements (Figure 4d). SAED patterns further confirmed the polycrystalline nature of the NiO, showing distinct diffraction rings indexed to the (111), (200), and (220) crystal planes (Figure 4e inset). High‐resolution TEM (HRTEM) images revealed that the crystalline structure of NiO was of short‐order characteristics. The lattice spacing of 0.241 nm corresponded to the (111) plane of crystalline NiO, while the surrounding amorphous regions were consistent with metallic Ru domains (Figure 4f,g). These microscopic observations aligned well with the XPS results, corroborating the conversion of RuO_2_ to metallic Ru during HER and the formation of a closely coupled metallic Ru/NiO interfacial structure, as schematically illustrated in Figure S13 (Supporting Information).

**Figure 5:**
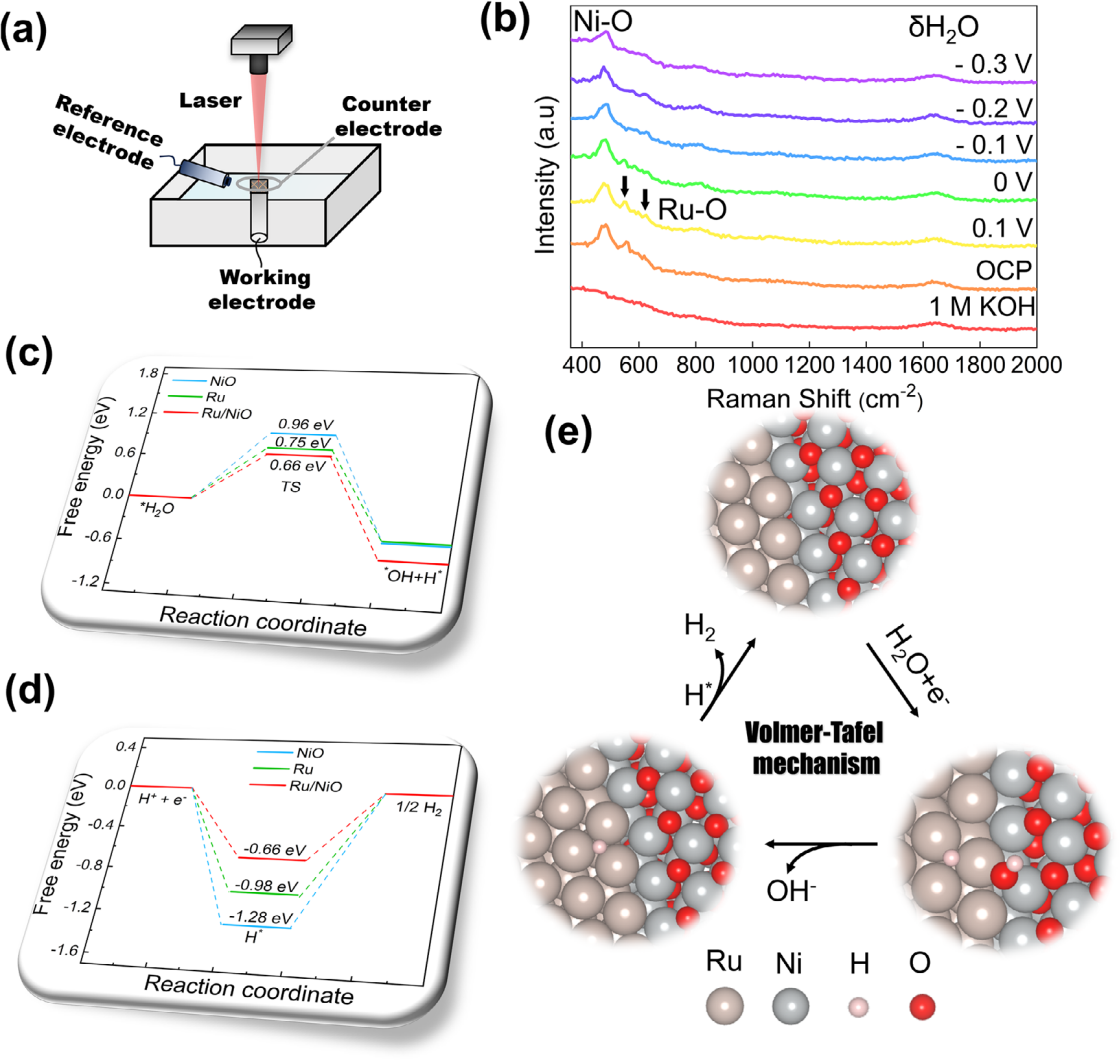
a) Schematic illustration of in situ electrochemical Raman system. b) In situ Raman spectra. c) Kinetic barrier of water dissociation on NiO (111), Ru (0001) and NiO (111)/Ru (0001) heterostructure, d) Calculated G_H*_ on different models, e) Schematic illustration of the HER reaction pathway on Ru (0001)/NiO (111) model.

To gain mechanistic insight into the structural evolution process, we performed in situ Raman spectroscopy to monitor the RuNiO_x_ catalyst under operational HER conditions. **Figure** **5**a,b displayed the in situ Raman spectra of the Ru/NiO catalyst acquired at various applied potentials, ranging from open circuit potential (OCP) to −0.3 V versus RHE. The peak located at 540 cm^−1^ was attributed to E_g_ vibrational modes of Ru─O and the peak at 626 cm^−1^ was assigned to the A_1g_ symmetric stretching mode of Ru─O in RuO_2_ (Noted by black arrow).^[^ ^54^, ^55^, ^56^ ^]^ Most notably, these two peaks reduced significantly as the potential reaches −0.1 V and disappears at −0.3 V, providing direct evidence for the electrochemical reduction of RuO_2_ to metallic Ru under cathodic conditions. In contrast, the Raman band at 490 cm^−1^, attributed to the Ni─O vibration, remained stable across the applied potentials.^[^ ^54^, ^57^ ^]^ This indicated that the NiO support maintained its stability throughout the HER process. A broad peak observed ≈ 1642 cm^−1^ was attributed to the δ(H─O─H) bending mode of water molecules.^[^ ^58^ ^]^ Therefore, the in situ spectroscopic evidence confirmed that the dynamically formed metallic Ru/NiO was active phases underpinning the enhanced HER activity.

Based on above results, DFT calculations were further employed to deepen the understanding of the catalytic mechanism of HER. Three models were established: the pristine NiO (111), Ru (0001), and Ru (0001)/NiO (111) heterogeneous interface according to the identified structures of these materials. In alkaline media, the cathodic hydrogen evolution reaction pathway necessitates an initial water dissociation process (Volmer step) succeeded by a hydrogen recombination step (either Heyrovsky or Tafel step). The inherently sluggish kinetics associated with the dissociation step critically impede the overall efficiency of water electrolysis.^[^ ^59^ ^]^ Consequently, the kinetic energy barrier of water dissociation was calculated. The simulated structure of the water dissociation process on the Ru (0001)/NiO (111) interface was depicted in Figure S14 (Supporting Information). The results showed that the energy barriers for water dissociation on Ru (0001) and NiO (111) are 0.75 and 0.96 eV, respectively (Figure 5c). While the Ru (0001)/NiO (111) interface exhibited a relatively lower energy barrier for water dissociation (0.66 eV), indicating synergistic effects at the interface. Apart from water dissociation, the descriptor, Gibbs free energy of adsorbed H* (G_H*_) is also important to evaluate HER performance and ideal electrocatalysts should have moderate G_H*_ of 0 eV.^[^ ^60^ ^]^ Figure 5d presented the calculated values of △G_H*_ for three models. Both pristine NiO (111) and Ru (0001) surfaces had △G_H*_ of −1.28 and −0.98 eV, respectively, too strong to desorb H from active sites. An expected outcome of coupling Ru (0001) with NiO (111) was a substantial reduction of H^*^ sorption strength (△G_H*_ = −0.66 eV), facilitating the conversion of H^*^ to H_2_ and accelerating the desorption of H_2_. Figure 5e displayed the schematic illustration of the HER reaction pathway on Ru (0001)/NiO (111) model. A water molecule (H_2_O) diffused to and adsorbed onto a heterogeneous interface. After accepting an electron (e^−^) from the electrode, the adsorbed water molecule underwent dissociation where OH^−^ tended to stabilize on the Ru (0001)/NiO (111) interface and H^*^ was prone to staying on the HCP hollow site of Ru (0001). Afterward, OH^−^ migrated into the bulk solution and absorbed H^*^ can combine with another H^*^ to yield molecular H_2_. In summary, the reconfigured Ru/NiO heterogeneous interface exhibited dual functional roles, simultaneously decreasing the energy barrier for H_2_O dissociation and optimizing the △G_H*_, resulting in favorable alkaline HER kinetics.

### 2.4 Seawater Electrolysis

**Figure 6:**
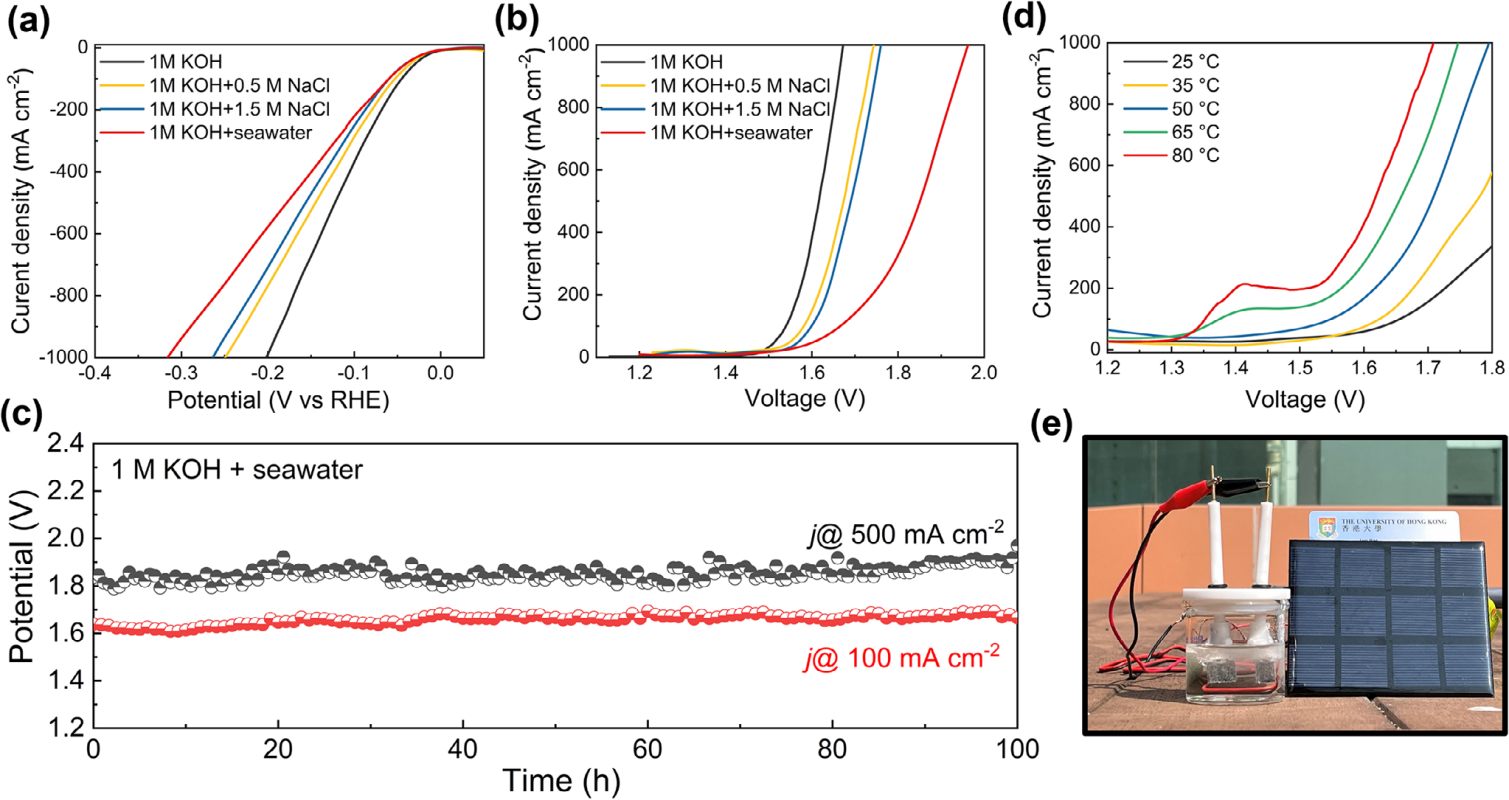
Polarization curves of a) RuNiO_x_ for HER in different electrolyte and b) two‐electrode alkaline water electrolysis in different electrolyte. c) Long‐term stability tests at constant current densities of 100 and 500 mA cm^−2^ in 1 m KOH + seawater. d) Polarization curves of S‐(NiFe)OOH // RuNiO_x_ for water splitting at different temperature in 1 m KOH + seawater. e) Pictures for hydrogen production of S‐(NiFe)OOH // RuNiO_x_ connected with the commercial silicon solar cell in 1 m KOH + seawater.

We further evaluated the HER performance of RuNiO_x_ electrode under industrial‐level high current densities in alkaline electrolyte, simulated alkaline seawater and alkaline seawater. In 1 M KOH electrolyte, the RuNiO_x_ electrode demonstrated exceptional performance, achieving overpotentials of 123 mV at 500 mA cm^−2^ and 204 mV at 1000 mA cm^−2^ (**Figure** **6**a). Furthermore, in simulated alkaline seawater with NaCl, the electrode maintained low overpotentials of 50 mV at 100 mA cm^−2^, 145 mV at 500 mA cm^−2^, and 253 mV at 1000 mA cm^−2^ in 0.5 m NaCl, along with 52, 156, and 262 mV at the same current densities in 1.5 m NaCl. The minor decrease in HER performance in electrolytes containing Cl^−^ is primarily attributed to the competitive adsorption of Cl^−^ ions, which may occupy active sites otherwise available for the hydrogen evolution reaction.^[^ ^61^, ^62^ ^]^ Even under more demanding conditions in alkaline seawater (1 m KOH + seawater), RuNiO_x_ still exhibited remarkable performance, with an overpotential of 59 mV (100 mA cm^−2^), 171 mV (500 mA cm^−2^), and 304 mV (1000 mA cm^−2^), respectively (Figure S15, Supporting Information). Above results exceeded most recent seawater electrocatalysts for HER, such as Ru‐NiMoO(P)_4_,^[^ ^39^ ^]^ Mo‐Ru/CNTs,^[^ ^63^ ^]^ and NC‐CoNi_2_S_4_@ReS_2_/CC (Table S2).^[^ ^64^ ^]^ We further attempted to perform the HER test using real seawater (pH 7.85) as the electrolyte (Figure S16a,b, Supporting Information). It is well‐known that natural water contains multiple detrimental species (Na^+^, Ca^2+^, Mg^2+^, Cl^−^, SO_4_^2−^, etc.) that would degrade the catalytic performance of an electrolyzer by passivating the electrode surface.^[^ ^65^ ^]^ In our test, by intermittently refreshing the seawater and cleaning precipitates on the electrode surface, the performance was essentially recovered under such harsh conditions (Figure S16c, Supporting Information). We also envisage that future work may focus on the development of a passivation layer to inhibit precipitates without frequent start‐shutdown seawater electrolysis to recover the electrode surface.

Inspired by the remarkable HER performance, we constructed a two‐electrode electrolyzer for overall alkaline seawater splitting by pairing an RuNiO_x_ cathode with a recent reported S‐(NiFe)OOH anode, the preparation and characterization of which were shown in the experimental section and Figure S17 (Supporting Information). The electrolyzer displayed excellent performance for overall seawater splitting in both simulated alkaline seawater and alkaline seawater electrolytes (Figure 6b; Figure S18, Supporting Information). In 1 m KOH electrolyte at 25 °C, the cell voltage required to achieve current densities of 100, 500, and 1000 mA cm^−2^ was only 1.540, 1.616, and 1.672 V, respectively. Moreover, it remained low in the presence of 1 m KOH + 0.5 m NaCl electrolyte, where applying 1.574, 1.672, and 1.742 V voltage yields current densities of 100, 500, and 1000 mA cm^−2^, respectively. As the concentration of NaCl increased in the electrolyte, a slight increase in cell voltage was observed to achieve the same current density. The cell voltage required to complete 100, 500, and 1000 mA cm^−2^ in 1 M KOH + 1.5 M NaCl electrolyte was 1.592, 1.688, and 1.759 V, respectively. Despite the observable attenuation of HER performance in alkaline seawater due to impurity interference, the cell voltages required to achieve current densities of 100, 500, and 1000 mA cm^−2^ were only 1.648, 1.874, and 1.958 V, respectively. These results remain superior to those of most reported seawater‐splitting electrocatalysts (Table S3), including Ru‐NiMoO(P)_4_,^[^ ^39^ ^]^ Ru‐CoO_x_/NF,^[^ ^66^ ^]^ and Ru‐Ni_2_P/Fe_2_P.^[^ ^67^ ^]^ We further evaluated the operational stability of the electrolyzer for alkaline seawater splitting under high current densities of 100 and 500 mA cm^−2^. As shown in Figure 6c, the cell voltage remained highly stable throughout continuous operation over 100 h at both current densities, demonstrating exceptional durability under industrially relevant conditions. To further corroborate the structural integrity and corrosion resistance of the catalyst, the electrolyte after stability test was collected and analyzed by ICP‐OES. The minimal leaching concentrations of Ru and Ni (7.60 and 2.14 µg L^−1^, respectively) confirm outstanding stability of the catalyst even in chloride‐containing seawater electrolyte. It was also reported that the incorporation of Ru species can significantly enhance the anti‐corrosion properties and structural durability of electrocatalysts in seawater electrolytes.^[^ ^54^, ^68^ ^]^

In an industrial alkaline water electrolysis system, the typical operational temperature ranges from 50–80 °C, which can further lower the cell voltage by reducing the kinetic barriers and elevating the ionic conductivity. We also mimicked similar operation conditions to further assess the practical potential of the electrolyzer. With increasing temperature, the cell voltage gradually lowered as kinetics boosted (Figure 6d). To achieve a current of 500 mA cm^−2^, the cell voltage was 1.71 V at 50 °C, 1.66 V at 65 °C, and 1.62 V at 80 °C, respectively. Meanwhile, the electrolyzer operated stably for 12 h at a temperature of 65 °C and a current density of 500 mA cm^−2^ (Figure S19, Supporting Information). Finally, a demo of a solar‐driven alkaline seawater splitting device was constructed, the power of which was supplied by a commercial silicon solar cell (Telesky, 5 V, 230 mA). As depicted in Figure 6e and Video S1 (Supporting Information), ample hydrogen bubbles were generated in the electrolyzer and thus renewable solar energy was stored to clean hydrogen gas.

## 3 Conclusions

In summary, this work showed a robust and highly efficient monolithic RuNiO_x_ electrode on nickel foam for alkaline HER. The electrode demonstrated outstanding performance at the industrial‐level current density of 1000 mA cm^−2^, requiring a low overpotential of 204 mV and maintaining exceptional stability for over 350 h. The combination of multiple complementary techniques, including in situ Raman spectroscopy, XPS, STEM, collectively confirmed that during the cathodic HER, RuO_2_ was reduced to metallic Ru, while NiO remained stable. This electrochemically derived Ru/NiO interface was identified as the key active center, where DFT simulations revealed an optimized adsorption affinity for key intermediates, synergizing with its altered hydrophilic–aerophobic surface to enhance both reaction kinetics and structural durability. When integrated with an efficient S‐(NiFe)OOH anode, the electrolyzer also showed excellent operational stability in alkaline seawater at high current densities. This rationally synthesized structure demonstrated a notable advancement toward creating a durable and efficient catalytic electrode for utilizing the vast seawater reserves of the planet to produce hydrogen on a large scale powered by renewable energy sources.

## Conflict of Interest

The authors declare no conflict of interest.

## Supporting information



Supporting Information

Supplemental Video 1

## Data Availability

The data that support the findings of this study are available from the corresponding author upon reasonable request.
