# Supplementary material for: Reconstructed Monolithic RuNi Heterostructure Enables Hydrogen Production from Alkaline Seawater at Industrial Current Density
Source: Adv Sci (Weinh). 2025 Oct 6;12(48):e10916. doi: 10.1002/advs.202510916 (PMC12752561; doi:10.1002/advs.202510916)
Supplement: Supplementary file 1 — Supporting Information [file ADVS-12-e10916-s002.docx]

Supporting Information

Reconstructed monolithic RuNi heterostructure enables hydrogen production from alkaline seawater at industrial current density

Hao Luo, Tao Zhou, Ruiqin Xia, Zhengxiao Guo*

Department of Chemistry, The University of Hong Kong, Hong Kong SAR

Email: zxguo@hku.hk

**Experimental section**

**Materials**

Ruthenium chloride hydrate (RuCl_3_·xH_2_O), iron nitrate nonahydrate (Fe(NO_3_)_3_·9H_2_O), nickel nitrate hexahydrate (Ni(NO_3_)_2_·6H_2_O), sodium thiosulfate pentahydrate (Na_2_S_2_O_3_·5H_2_O), potassium hydroxide (KOH), and sodium chloride (NaCl) were purchased from Aladdin. Ruthenium oxide (RuO_2_), 5 wt.% Nafion solution, 20 wt.% Pt on Vulcan carbon, and anhydrous ethanol (≥99.5%) were purchased from Sigma-Aldrich. All materials are received as use without further purification. Deionized (DI) water was obtained from the Milli-Q water purification system at an electrical resistivity of 18.2 MΩ·cm. Real seawater (pH value: ~7.85) was collected in Kennedy Town, Hong Kong Island and filtered to remove macroscopic sediments. Ni foam was purchased from Suzhou Keshenghe Metal Product Co., Ltd. with pore per inch (PPI) of 110 and thickness of 1 mm.

**Solvothermal Synthesis of the RuNiO_x_ electrode:**

The solvothermal synthesis of RuNi precursor was referred to previous literature with the major modification.^[1]^ First, nickel foam was cut into pieces with the size of 2 cm × 3 cm in area and then sonicated in concentrated HCl for 5 mins to remove the surficial oxidation layer, followed by washing with water and ethanol three times. Second, nickel foam and 10 mM RuCl_3_·xH_2_O ethanol solution were added into 50 mL Teflon-lined stainless-steel autoclave, maintaining 80℃ for 8 hours. Finally, the electrode was subjected to thermal annealing in air at either 300 °C or 500 °C for 4 hours with heating rate of 1 ℃ min^–1^. The final electrode was named RuNiO_x_. NiO electrode supported on Ni foam was fabricated in the same way by using 10 mM Ni(NO_3_)_2_·6H_2_O ethanol solution for the solvothermal reaction.

**Preparation of commercial RuO_2_ (C-RuO_2_) and Pt/C on nickel foam:**

5 mg of commercial RuO_2_ was dispersed in 1 mL of a mixed solvent consisting of 970 µL ethanol and 30 µL of 5 wt.% Nafion solution, followed by sonication for 3 hours to form a homogeneous ink. Similarly, a Pt/C-based ink was prepared by dispersing 5 mg of 20 wt.% Pt/C in the mixed solvent composition (970 µL ethanol and 30 µL of 5 wt.% Nafion solution) and sonicating for 3 hours. Then, 200 µL of each catalyst ink was dip-coated onto nickel foam substrates and dried at room temperature to obtain the C-RuO₂ and Pt/C electrodes, respectively. The total catalyst loading was controlled at ~1 mg_total_ cm^–2^ for both electrodes.

**Preparation of** **S-(NiFe)OOH on nickel foam:**

The preparation of S-(NiFe)OOH on nickel foam was referred to the literature.^[2]^ Typically, 2 cm×3 cm Ni foam was immersed in an aqueous solution containing 35 g L^–1^ Fe (NO_3_)_3_·9H_2_O and 5 g L^–1^ Na_2_S_2_O_3_·5H_2_O. After 3 mins, the sample was taken out and washed with DI water three times and dried at room temperature.

**Characterizations:**

The field emission scanning electron microscope was observed by TESCAN MAIA3 XMH. The powder X-ray diffraction spectra were acquired on high brilliance X-ray diffractometer Bruker D8 ventre with Cu Kα radiation (λ=1.5418 Å). The transmission electron microscopy and scanning transmission electron microscopy were obtained on JEM-2100F, JEOL. The X-ray photoelectron spectroscopy was carried out by Thermo Scientific K-Alpha with the excitation source of Al K-alpha ray (HV=1486.6 eV). The contact angle test was conducted on Dataphysics, OCA-20. The optical image of hydrogen bubble generation was recorded on the Leica Flexacam c5 microscope using a two-electrode configuration under a constant current of 500 mA cm^–2^. In-situ Raman spectroscopy was performed on a Horiba LabRAM HR Evol confocal microscope with excitation from a 532 nm argon ion laser. RuNiO_x_ was served as the working electrode, Pt wire as the counter electrode and Hg/HgO as the reference electrode. In-situ Raman spectra were acquired in 1 M KOH at fixed potentials using chronoamperometry with a CHI760E electrochemical workstation. The elemental composition was characterized by Inductively Coupled Plasma Optical Emission Spectrometry (ICP-OES, Agilent 5110). A portion of the electrode was digested using a microwave-assisted system (Multiwave 3000, Anton Paar) to obtain a homogeneous solution for analysis.

**Electrochemical test**

Electrochemical tests were conducted on the CHI760E electrochemical workstation using a three-electrode system with different alkaline electrolytes (1 M KOH, 1 M KOH + 0.5 M NaCl, 1 M KOH + 1.5 M NaCl, 1M KOH + seawater, real seawater). Self-supported Ni foam loading with catalysts were served as the working electrode, whereas a Hg/HgO electrode as the reference electrode and carbon rod electrode as the counter electrode. All potentials in this work were calibrated to the reversible hydrogen electrode (RHE) by the following equation 1:

E_RHE_ = E_Hg/HgO_ +0.098+0.059 pH (equation 1)

Before each test, the electrolyte was bubbled by argon gas for 30 min and purged by argon gas during the test. Electrodes were first cycled at 0 to –0.6 V (vs. RHE) for cycles until curves coincide. Afterwards, the polarization curves of different samples were tested using linear scan voltammetry (LSV). The reaction kinetics were deduced from Tafel plots derived from the LSV curves using the Tafel equation: η=a+blog(|j|). Cyclic voltammetry was used for calculating the electrochemical double-layer capacitance C_dl_. The linear relationship between the scan rate (v) and the mid-point current density (Δj) was established in the non-faradaic region by at different scanning speeds of 10, 30, 50, 70, and 90 mV s^–1^. The stability test was performed by comparing voltage change against time using the Chronopotential method under a fixed current densities. The volume of hydrogen produced by water electrolysis was measured by the water-gas displacing method at a current density of 20 mA cm^-2^. The Faradaic efficiency of hydrogen production was then calculated using equation 2, where the theoretical gas volume is given by equation 3,

V$\text{=}\frac{\text{Q}}{\text{zF}}$ × $\frac{\text{RT}}{\text{P}}$ (equation 2)

Faradic efficiency %= $\frac{\text{V}_{\text{Measured value}}\text{ }}{\text{V}_{\text{Theoretical value}}}$ ×100% (equation 3)

Where Q was the charge produced by the electrochemical reaction, which was equal to the result of current (i) and time (t). R was the ideal gas constant (8.314 J mol^–1^ K^–1^), T was the thermodynamic temperature (298.15 K), F was the Faraday constant (96485 C mol^–1^), Z was the number of electrons transferred during the reaction (2 for hydrogen evolution), and P was the standard atmospheric pressure (101325 Pa). Two-electrode water splitting system was conducted either powered by electricity or solar panel-generated electricity, whereas the S-NiFeOH served as the anode and the RuNiO_x_ acted as the cathode. The polarization curve was scanned over a range of 1-1.8 V at a scanning rate of 1 mV s^–1^. Without a specific note, all the electrochemical measurement was performed at room temperature ~25 °C and the electrochemical data was corrected with 85 % iR compensation.

**Calculation details**

All the spin-polarized DFT calculations were performed by the Vienna Ab initio Simulation Package (VASP) using the projector augmented wave (PAW) method.^[3-4]^ The exchange-functional was treated using the generalized gradient approximation (GGA) with Perdew-Burke-Ernzerhof (PBE) functional.^[5]^ The energy cutoff for the plane wave basis expansion was set to be 500 eV and DFT-D3 was incorporated to describe the van-der-Waals interactions. The convergence criterion of energy and Hellmann-Feynman force were set to be smaller than 10^–4^ eV and 0.05 eV Å^–1^, respectively. The lattice parameter of NiO bulk structure was optimized using the k-mesh of 4×4×4. The NiO (111) surface with the O termination was built, where the k-mesh of 2×2×1 was used in all the calculations. Ru (0001) surface with 4 layers was built from the bulk structure of Ru. The heterogeneous interface model was built by adopting NiO (111) surface and Ru (0001) surface with lattice mismatch below 5%. A 15 Å vacuum layer was introduced along the Z-axis to avoid mirror interactions between two adjacent layers. The free energy for electrochemical steps was calculated from equation 4 based on the computational hydrogen electrode (CHE) model.^[6-7]^

ΔG = ΔE + ΔE_ZPE_ – TΔS (equation 4)

where ΔE, ΔE_ZPE_ and ΔS referred to the DFT calculated energy change, the change of zero-point energy, the change in entropy of products and reactants, respectively. The free energy corrections were considered at the temperature of 298.15 K.

**
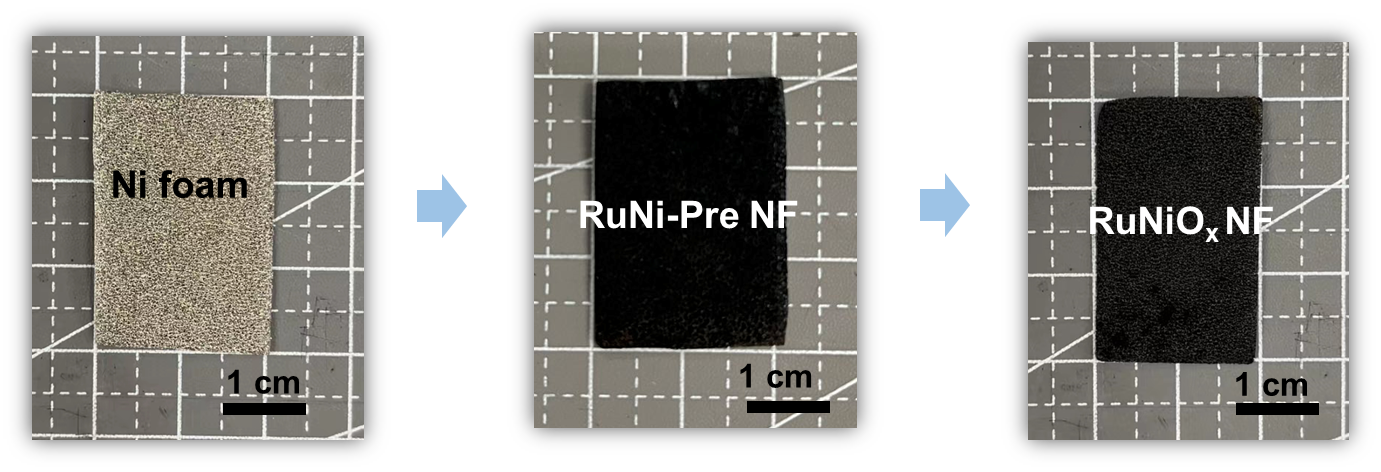
**

**Figure S1.** Optical images of neat NF, RuNi-Pre_,_ and RuNiO_x._

The selected solvothermal conditions (80 °C, 8 h) enabled the successful deposition of a RuNi precursor layer, which is uniformity proved critical to the electrocatalytic performance, as it ensured the formation of a well-integrated active phase after annealing. An insufficient or non-uniform precursor would have resulted in poor electrical connectivity and mechanical instability, particularly under high-current-density operation.

**
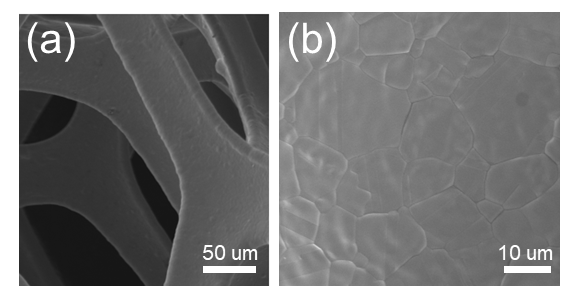
**

**Figure S2.** SEM images of neat Ni foam with different magnifications.


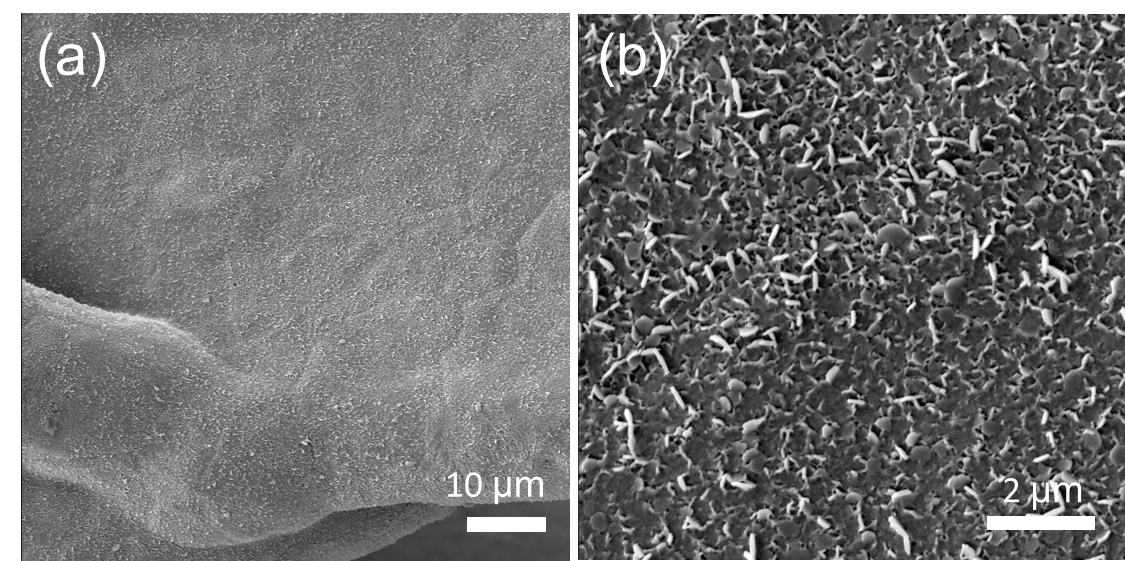


**Figure S3****.** SEM images of NiO with different magnifications.


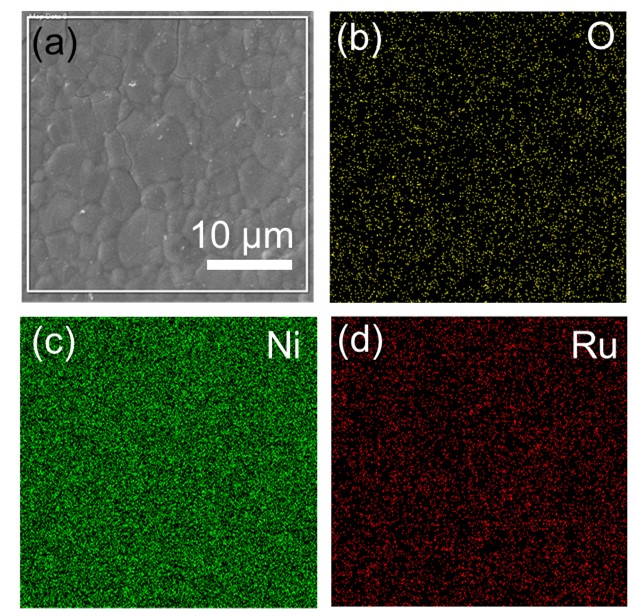


**Figure S4.** Element mapping images of RuNiO_x_ electrode.


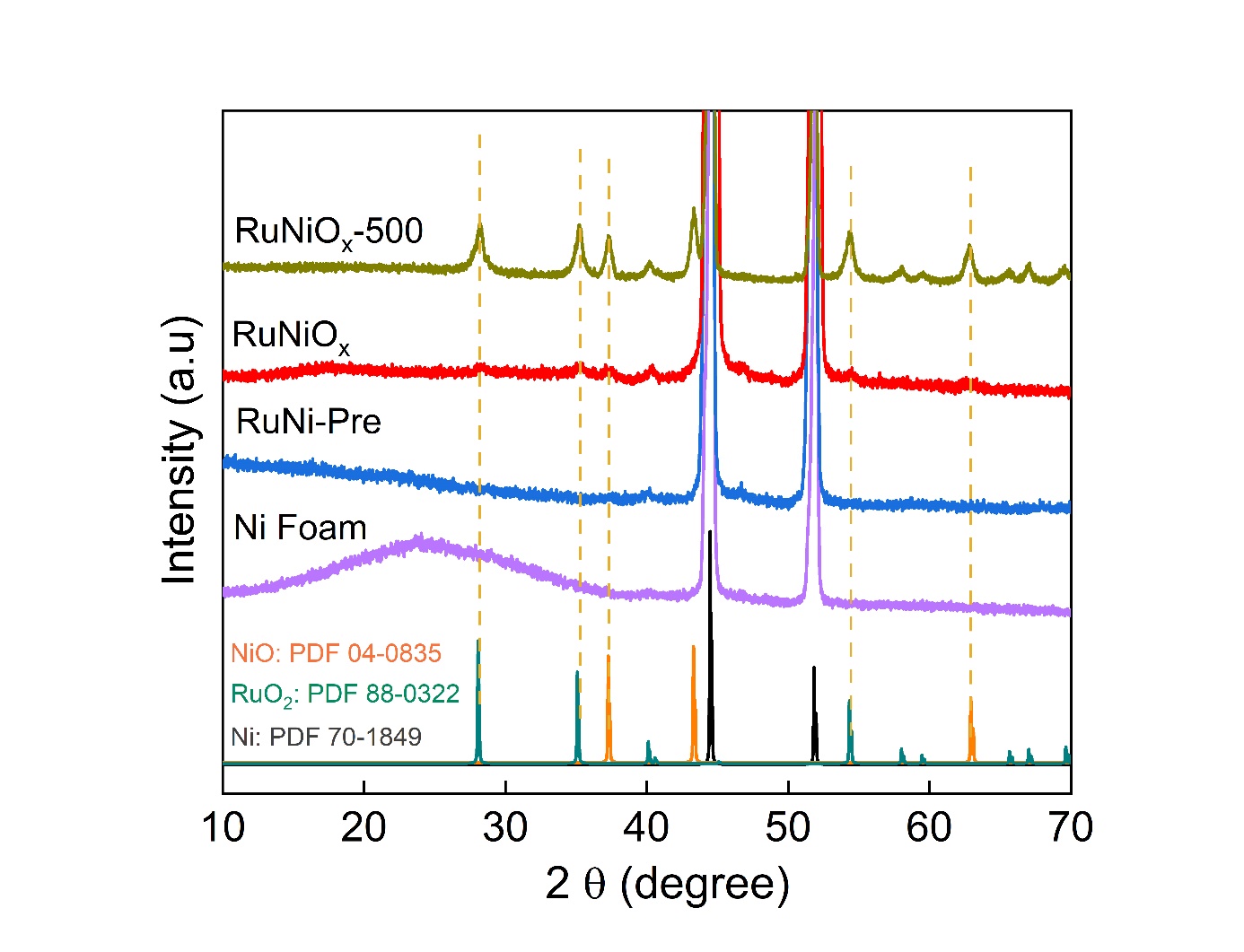


**Figure S5.** XRD pattern of NF, RuNi-Pre, RuNiO_x_, and RuNiO_x_-500.


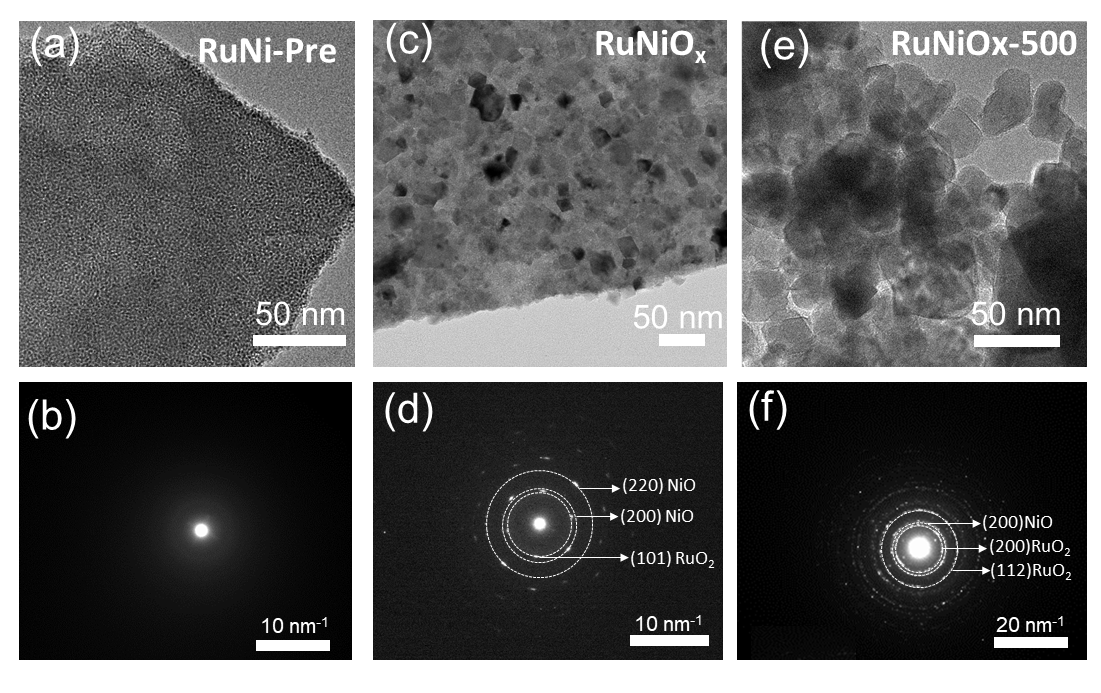


**Figure S6.** TEM images and SAED of (a, b) RuNi-Pre, (c, d) RuNiO_x_, and (e, f) RuNiO_x_-500.


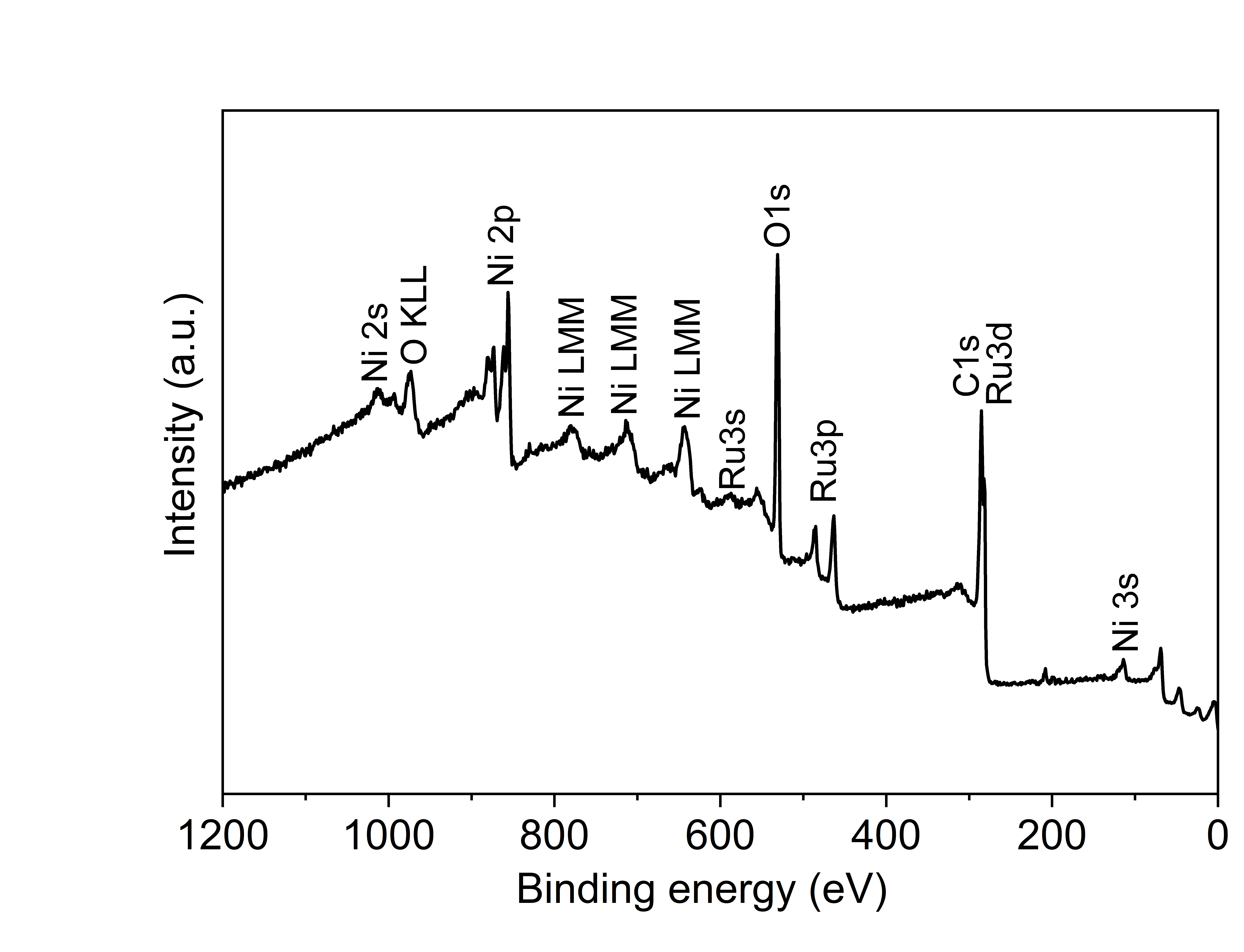


**Figure S7.** XPS survey spectra of the RuNiO_x_.


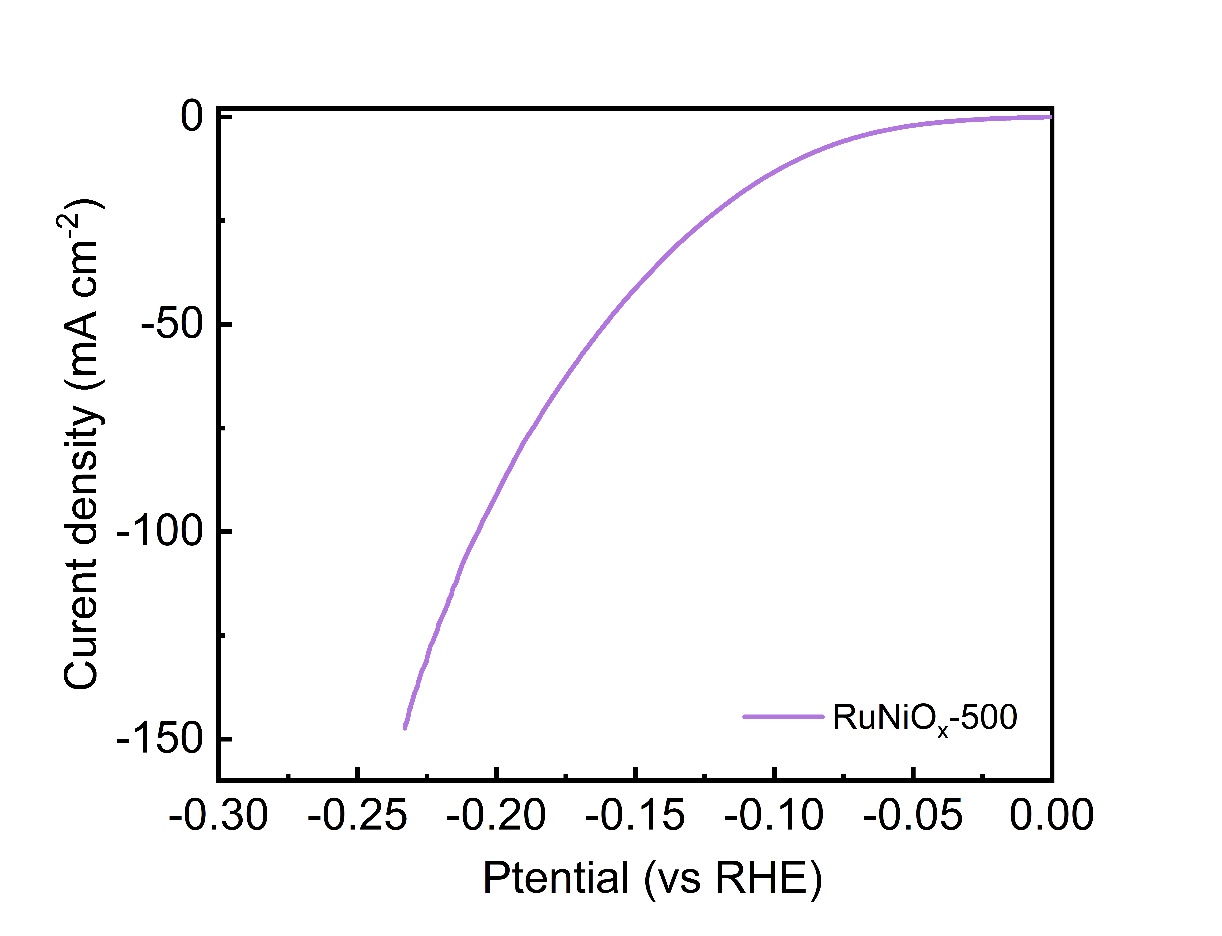


**Figure S8**. Polarization curves of RuNiO_x_-500 measured in 1 M KOH.

As shown in Figure S8, to deliver current densities of 10, 50, and 100 mA cm^–2^, the RuNiO_x_-500 electrode need overpotentials of 91, 161, and 207 mV respectively. RuNiO_x_-500 exhibited inferior electrocatalytic performance compared to RuNiOₓ, which can be attributed to severe nanograin agglomeration caused by the high annealing temperature.


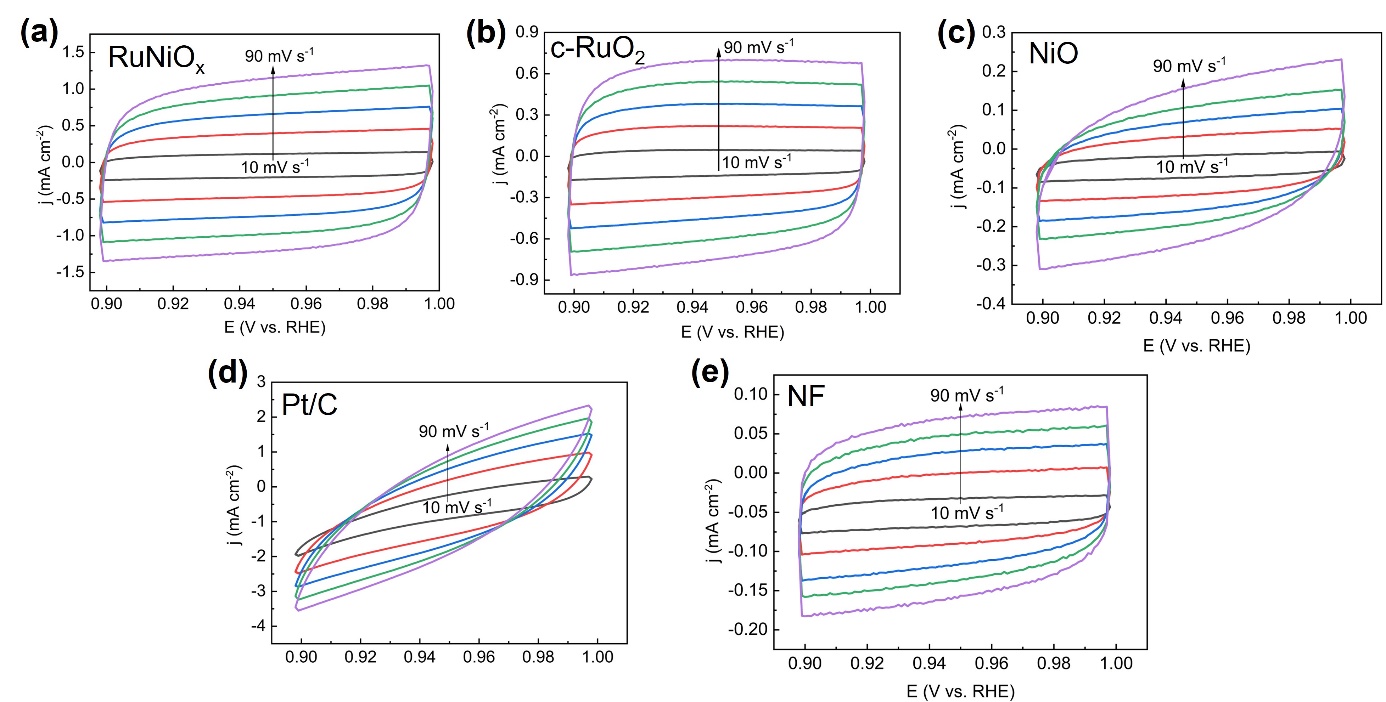


**Figure S9.** CV curves recorded within the non-faradic region in 1 M KOH electrolyte.


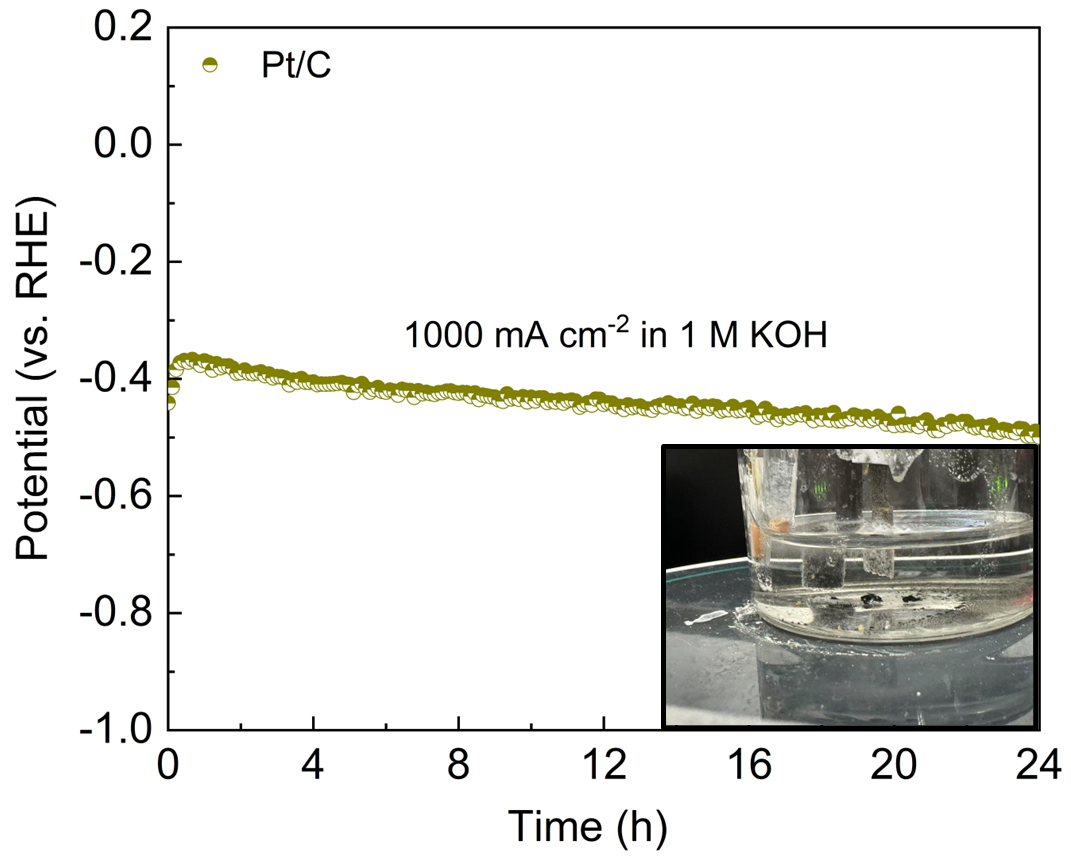


**Figure S10.** Stability test of Pt/C electrode in 1 M KOH electrolyte, inset: photograph of electrolyzer vessel after stability test.


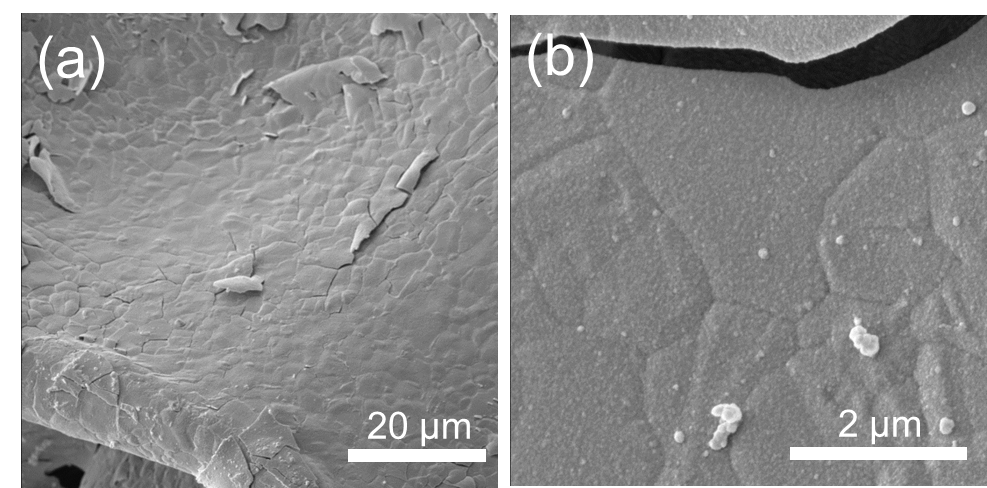


**Figure S11.** SEM images of RuNiO_x_ after long-term HER.


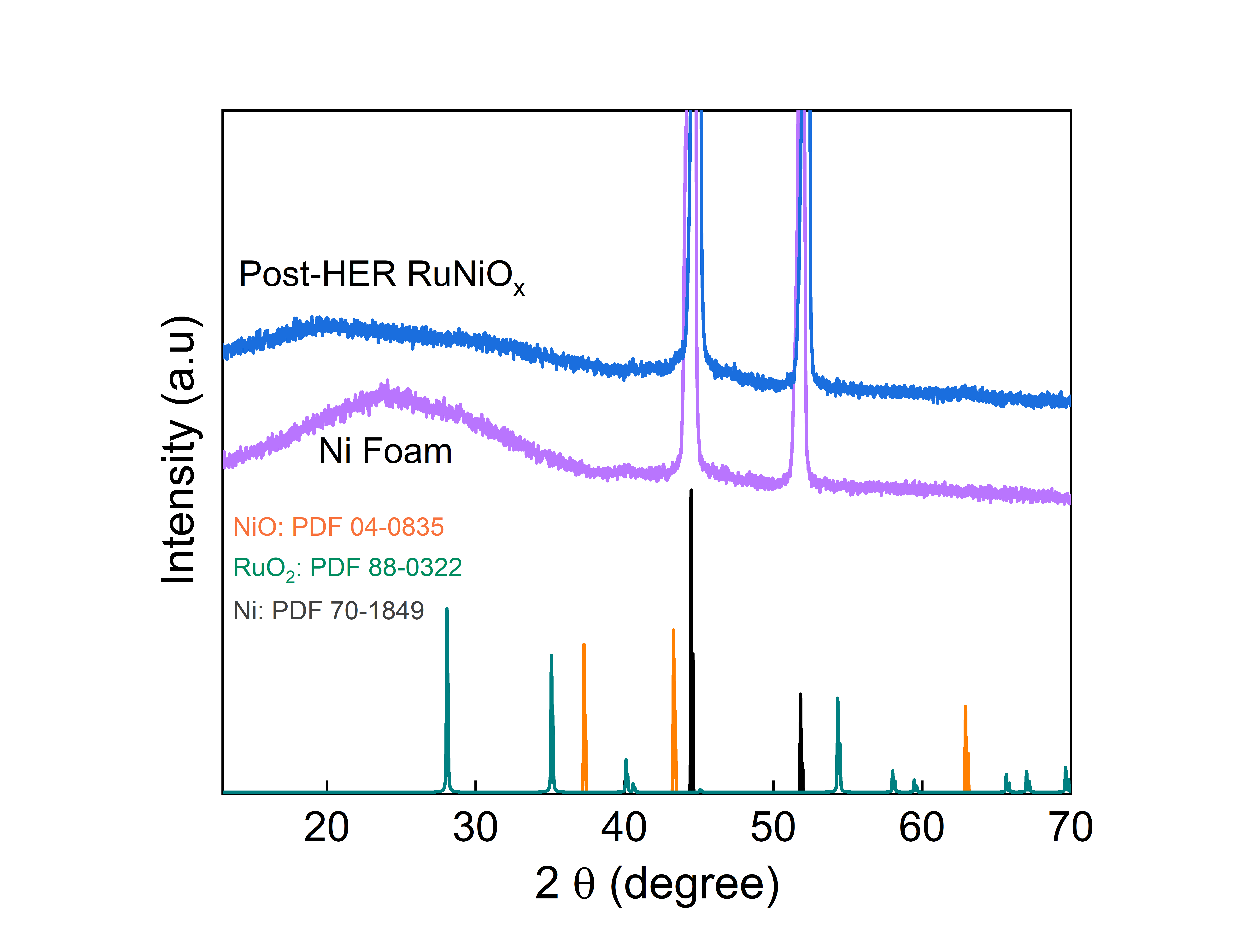


**Figure S12.** XRD pattern of the RuNiOₓ electrode after long-term HER at 1000 mA cm^–2^ for 350 hours.


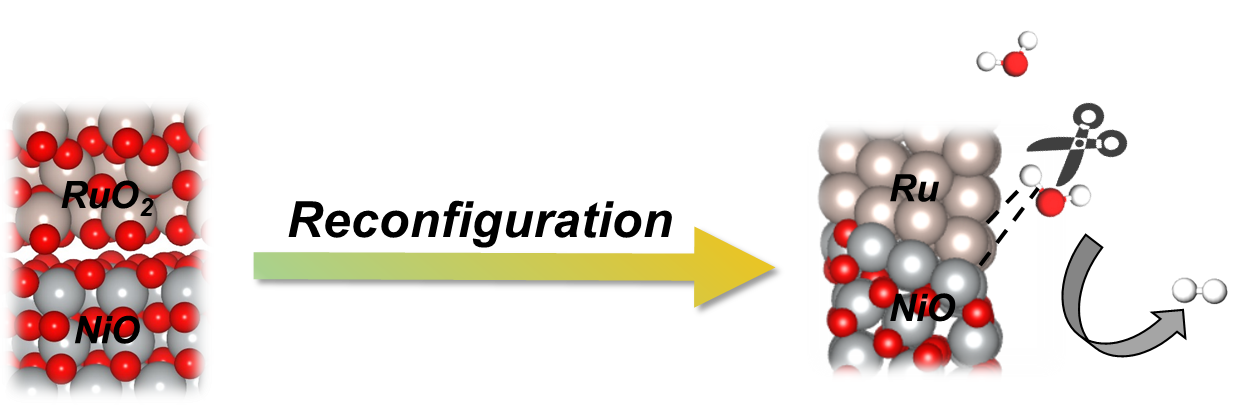


**Figure S13.** Schematic illustration for reconstruction of RuNiO_x_ during HER process.


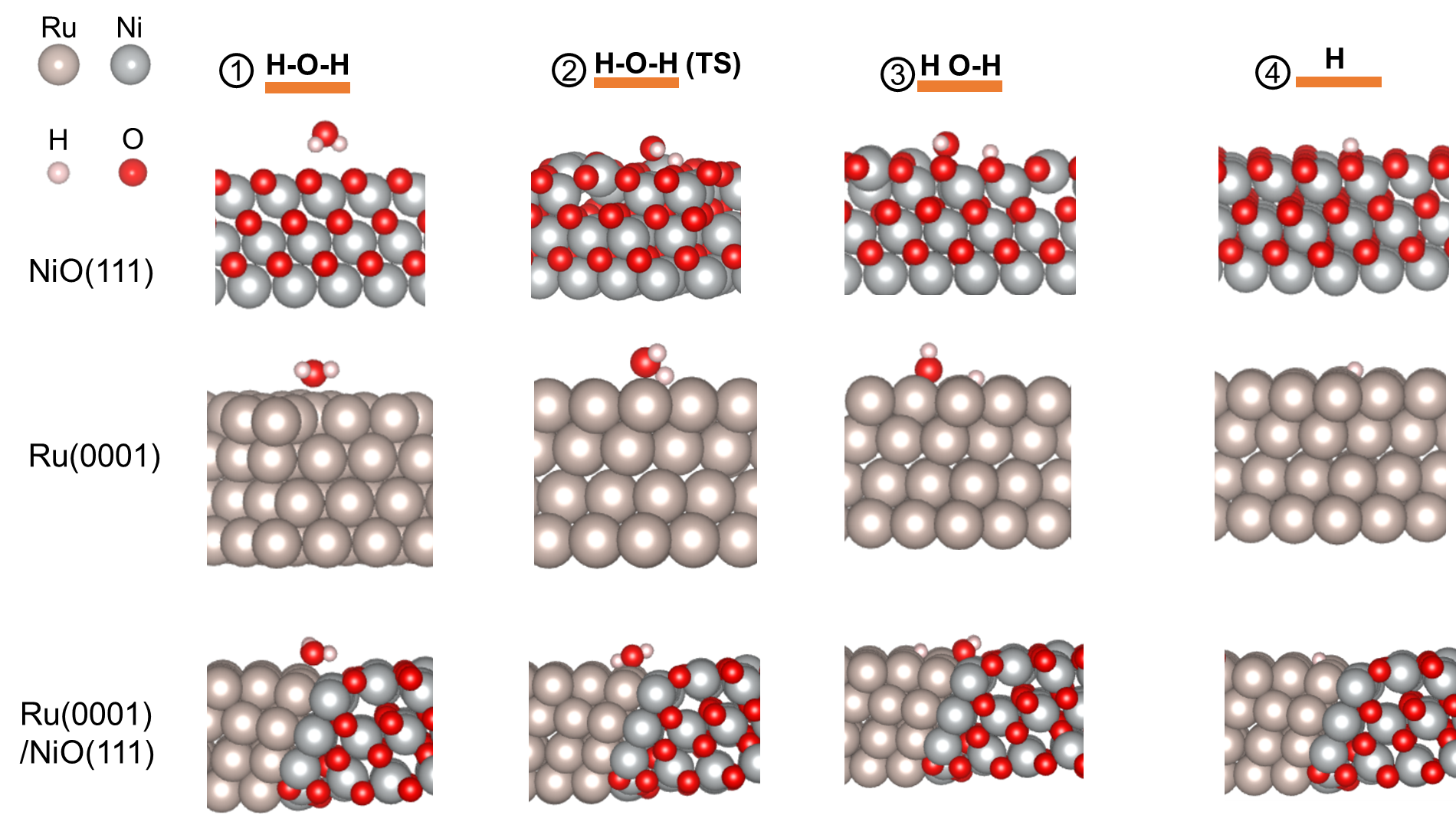


**Figure S14.** Side view of optimized models of water dissociation and hydrogen adsorption processes on different surfaces.


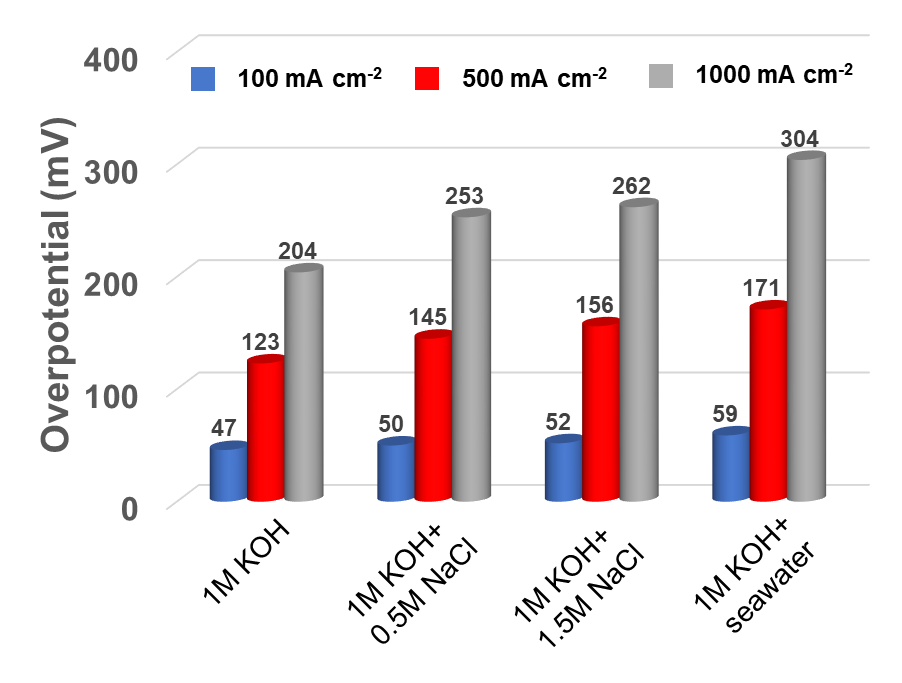


**Figure S15.** Comparison of overpotentials at 100, 500,1000 mA cm^–2^ in different electrolytes.


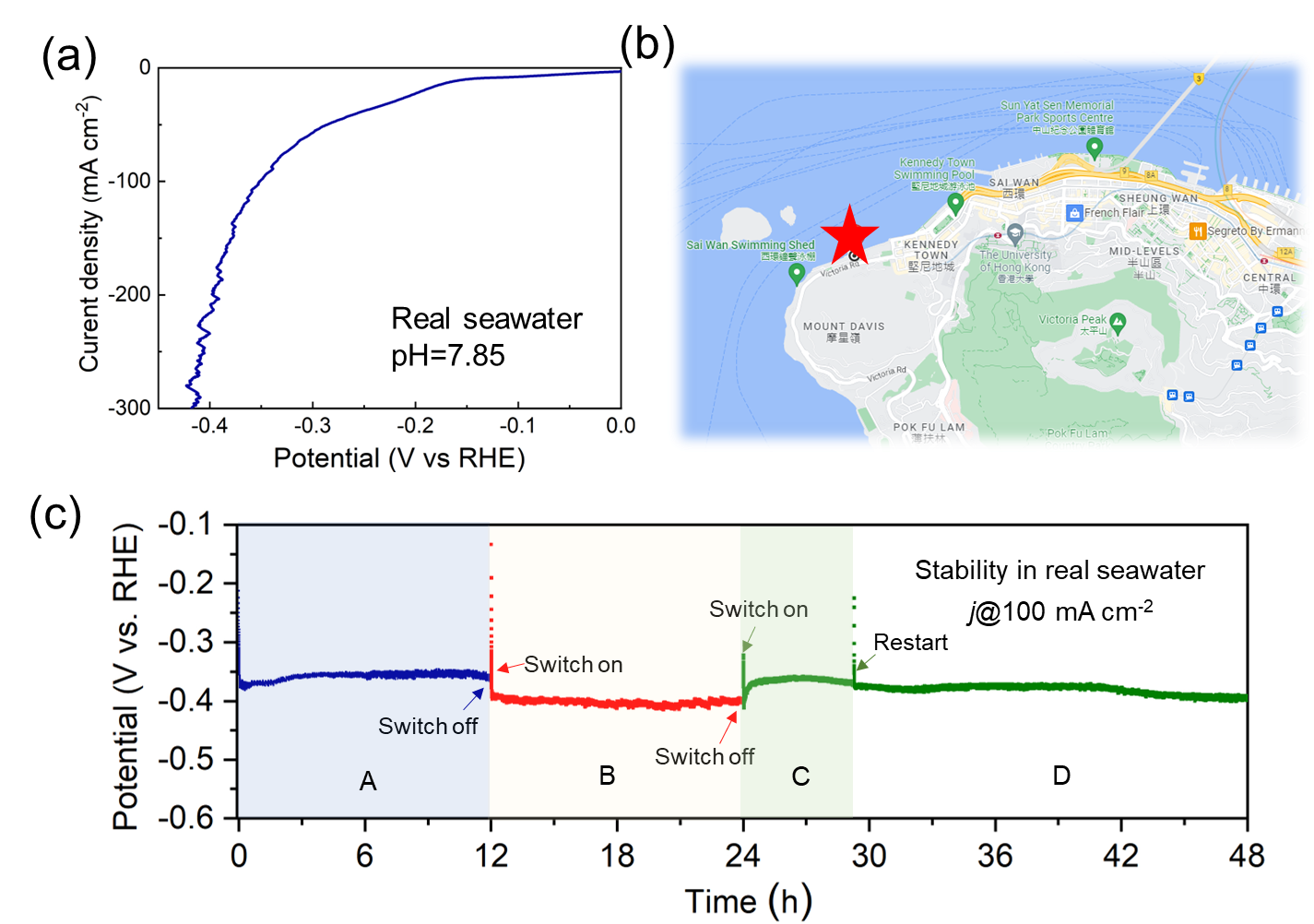


**Figure S16.** (a) The HER polarization curves of RuNiO_x_ in natural seawater. (b) Specific position of used neutral seawater. (c) Stability tests at a constant current density of 100 mA cm^–2^ in natural seawater.

As shown in Figure S16a, RuNiO_x_ can yield a current of 100 mA cm^–2^ at a voltage of –0.35 V vs. RHE. When scanning towards the negative region, the curve fluctuated, and we also observed that a layer of white precipitate appeared on electrode surface. In the cathodic HER process, water molecules (H_2_O) were consumed to generate hydrogen (H_2_) and hydroxide ion (OH^–^). The local pH change will induce the precipitation of insoluble metal salts on electrode surface, as described in equation 5 and 6.

Mg²⁺(aq) + 2OH⁻(aq) → Mg(OH)₂(s) ↓ (equation 5)

Ca²⁺(aq) + HCO₃⁻(aq) + OH⁻(aq) → CaCO₃(s) ↓ + H₂O(l) (equation 6)

We also carried out the stability test of HER in above real seawater. In the first region (zone A), the voltage remains stable against time, yet the curve showed more or less fluctuation as the layer of precipitation may block the effective bubble release. We switched off the electricity and cleaned the electrode surface. Then, we resumed the test, but the voltage dropped suddenly and remained partially stable in the next 12 hours, indicating the performance worsened (Zone B). We assumed it came from the deterioration of electrolyte as it appeared turbidity during extended periods of seawater electrolysis. We switched off the electricity after 24 hours. Afterwards, we picked up the electrode to clean the surface and refilled a new bottle of electrolyte. The performance was recovered, and voltage was returned to the initial stage (Zone C). We swiftly switched off and switched on the electricity, the voltage coincided well with that of the last period test and did not increase considerably during the test (Zone D).


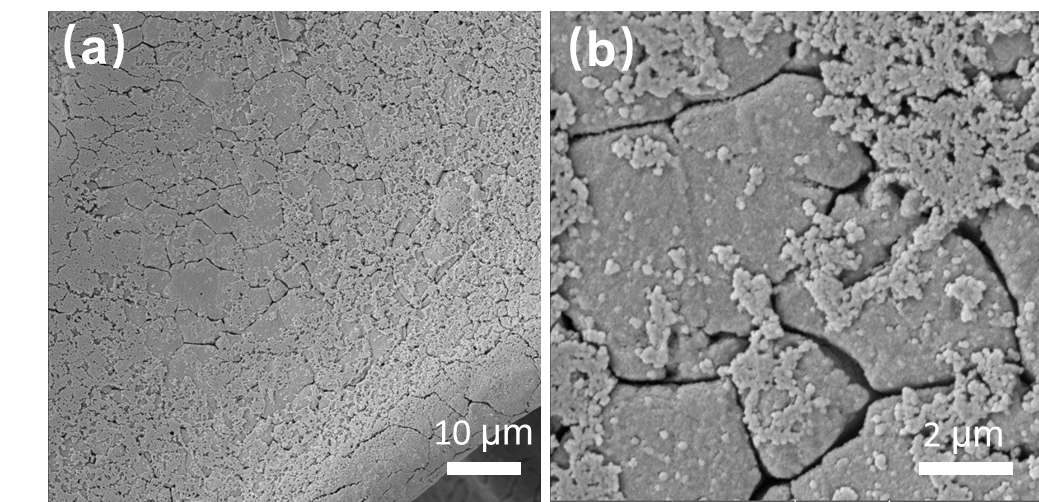


**Figure S17.** SEM images of S-(NiFe)OOH with different magnifications.


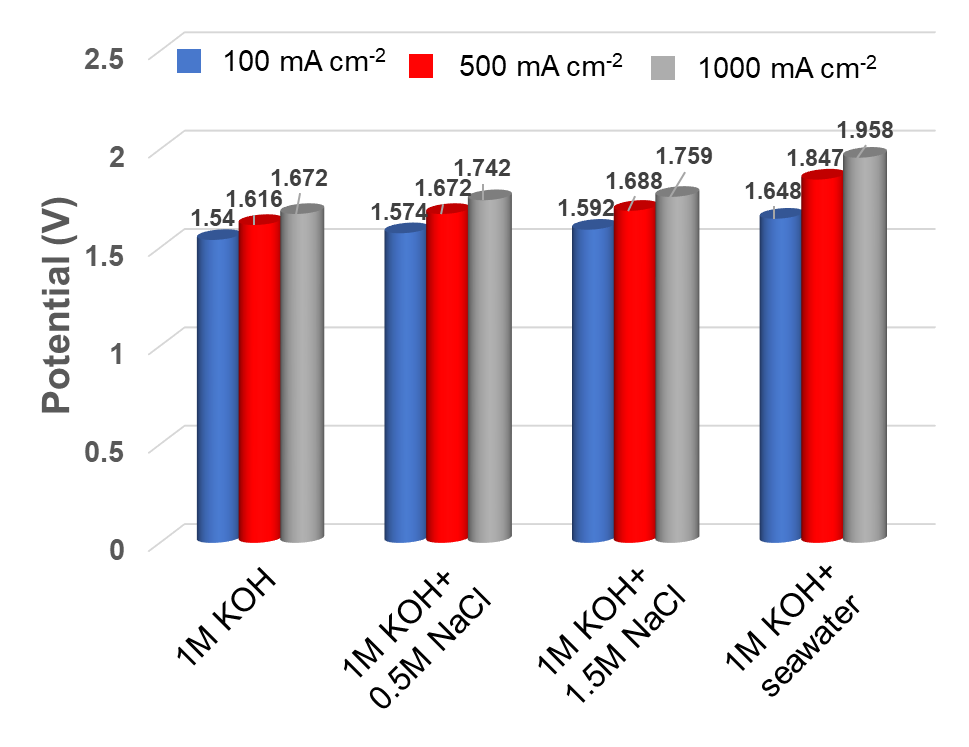


**Figure S18.** Comparison of cell voltage in different electrolyte using S-(NiFe)OOH //RuNiO_x_.


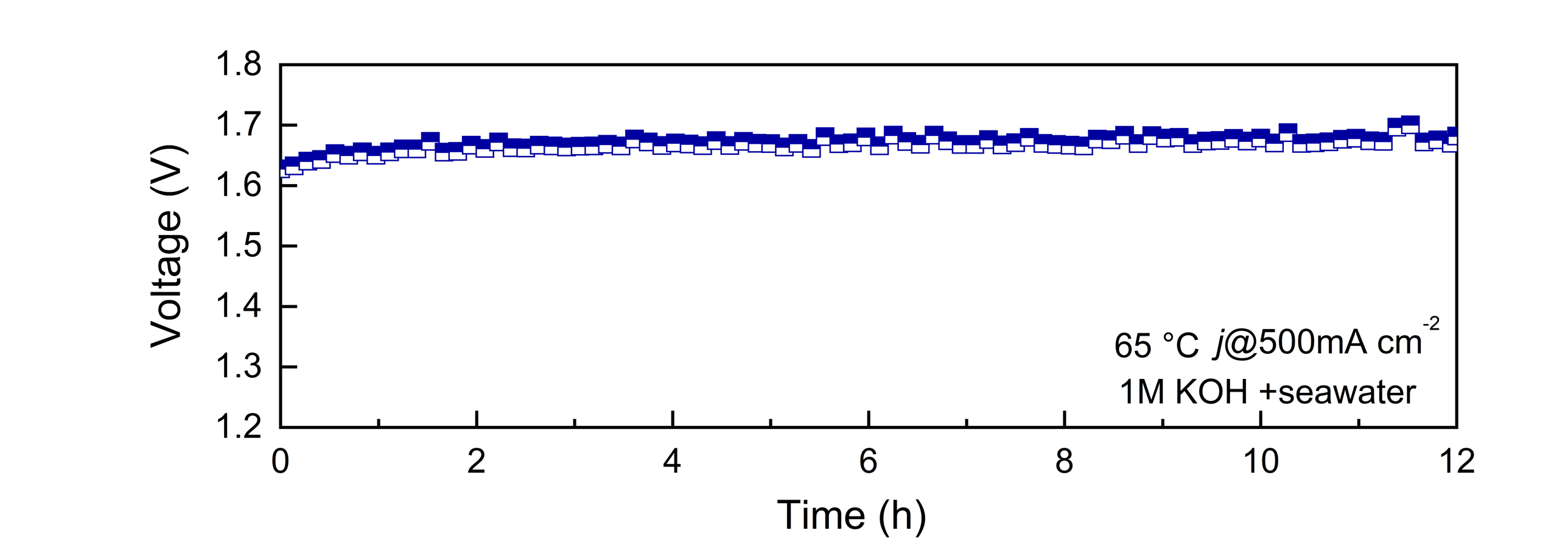


**Figure S19**. Stability tests at 500 mA cm^-2^ in 1 M KOH + seawater at a temperature of 65 °C.

**Note: Calculation of Mass Activity and cost-effective analysis**

*1. Calculation of Electrode Areal Density:*

We selected a piece of RuNiO_x_ electrode for ICP-OES test with dimensions:

22 mm × 12.5 mm = 2.2 cm × 1.25 cm = 2.75 cm²

Mass of the RuNiO_x_ electrode: 0.0845 g = 84.5 mg

Areal Density (Mass of electrode per unit area):
Areal Density = Total Mass / Geometric Area = 84.5 mg / 2.75 cm² ≈ 30.73 mg_electrode_ cm^–2^

*2. Calculation of Ru Mass Loading:*

The bulk composition of the electrode was determined by ICP-OES, revealing a Ru content of 0.00797 mg of Ru per mg of electrode.

Ru Mass Loading (mg_Ru_ cm^–^²):
Ru Loading = Areal Density × Ru Content = 30.73 (mg_electrode_ cm^–2^) × 0.00797 (mg_Ru_ / mg_electrode_), therefore, Ru _Loading_ ≈ 0.245 mg_Ru_ cm^–2^

*3. Calculation of Mass Activity:*

Current Density (j): The current density at an overpotential (η) of 50 mV was obtained from the LSV curve (Figure 2a). The value of this condition is 0.115 A cm^–2^.

Mass Activity (A mg^–1^_Ru_):
Mass Activity = j (A cm^–2^) / Ru Loading (mg_Ru_ cm⁻²) = 0.115 A cm⁻² / 0.245 mg_Ru_ cm^–2^ ≈ 0.47 A mg^–1^ _Ru_

*4. Comparison with Benchmark Pt/C Catalyst:*

Current Density (j): The current density at an overpotential (η) of 50 mV was obtained from the LSV curve (Figure 2a). The value of this condition is 0.0429 A cm^-2^. The mass loading of noble Pt is 0.2 mg_Pt_ cm^–2^.

Mass Activity (A mg^–1^_Pt_):
Mass Activity = j (A cm^–2^) / Pt Loading (mg_Ru_ cm^–2^) = 0.0429 A cm^–2^ / 0.2 mg_Pt_ cm^–2^ ≈ 0.21 A mg^–1^_Pt_

Fold Increase in Mass Activity:
Fold Increase = Mass Activity of RuNiO_x_ /Mass Activity of Pt/C = 0.47 A mg^–1^ / 0.21 A mg^–1^ ≈ 2.2

*5. Cost Analysis (Based on approximate spot prices from Apmex, Aug 2025):*

Ruthenium (Ru) Price: ~$935 USD/troy oz ≈ $30.06 per gram

Platinum (Pt) Price: ~$1369 USD/troy oz ≈ $44.01 per gram

Price Ratio: $30.06 / $44.01 ≈ 0.68
This indicates that Ru is approximately 68% the cost of Pt on a per-mass basis.

**Table S1** The comparison of our work with recent reported HER electrocatalysts in 1 M KOH electrolytes.

| Electrocatalysts | Overpotentials  (mV)@10 mA cm^–2^ | Overpotentials (mV)@50 mA cm^–2^ | Overpotentials (mV)@100 mA cm^–2^ | References |
| --- | --- | --- | --- | --- |
| RuNiO_x_ | 16 | 36 | 47 | This work |
| Ru-RuO_2_/C | 31 | 80 | N.A. | ^[8]^ |
| V-CoP@a-CeO_2_ | 68 | 115 | 140 | ^[9]^ |
| Ru-NiMoO(P)_4_ | 24 | 47 | 59 | ^[10]^ |
| Ru-Ni_3_N/NiO | N.A. | 46 | 63 | ^[11]^ |
| Pt1/(Co,Ni)(OH)_2_/C | 24 | 62 | 86 | ^[12]^ |
| Ru/Co_4_N/NF | 45 | 99 | 145 | ^[13]^ |
| RuNP-WN/CC | 9 | 19 | 22 | ^[14]^ |
| Sr_2_RuO_4_ | 18 | 43 | N.A. | ^[15]^ |
| Ru-Ni_3_Se_2_/FeSe_2_ | 43 | 90 | N.A. | ^[16]^ |
| RuO_X_ | 18 | 40 | 57 | ^[17]^ |
| Ru/NiMnB | N.A. | 103 | 141 | ^[18]^ |

**Table S2** The comparison of our work with recent reported HER electrocatalysts in alkaline seawater electrolytes (1 M KOH + seawater).

| Electrocatalysts | Overpotentials  (mV)@100 mA cm^–2^ | Overpotentials (mV)@500 mA cm^–2^ | Overpotentials (mV)@1000 mA cm^–2^ | References |
| --- | --- | --- | --- | --- |
| RuNiO_x_ | 59 | 171 | 204 | This work |
| Ru-NiMoO(P)_4_ | 117 | N.A. | N.A. | ^[10]^ |
| Mo–Ru/CNTs | 143.9 | N.A. | N.A. | ^[19]^ |
| NC-CoNi_2_S_4_@ReS_2_/CC | 200 | N.A. | N.A. | ^[20]^ |
| Co_0.07_Ce_0.93_O_2−δ_ | 309 | 474 | N.A. | ^[21]^ |
| NiCoMoPO | 275 | 404 | 470 | ^[22]^ |
| NiFeS/NF | 217 | 347 | N.A. | ^[23]^ |
| NiCoP foam/NF | 171 | 262 | 328 | ^[24]^ |
| NiMoN | 82 | 160 | 218 | ^[25]^ |
| NiPx@HA | 284 | 550 | N.A. | ^[26]^ |
| Ru/NiMoO_4-x_ | N.A. | N.A. | 170 | ^[27]^ |

**Table S3** Comparison of the potential with the reported overall alkaline seawater splitting catalysts. (1 M KOH + seawater).

| Electrocatalysts | Overpotentials  (mV)@100 mA cm^–2^ | Overpotentials (mV)@500 mA cm^–2^ | Overpotentials (mV)@1000 mA cm^–2^ | References |
| --- | --- | --- | --- | --- |
| S-(Ni, Fe)OOH //RuNiO_x_ | 1.648 | 1.874 | 1.958 | This work |
| NiCoMoPO | 1.850 | 2.096 | N.A. | ^[22]^ |
| NC-CoNi_2_S_4_@ReS_2_/CC | 1.83 | N.A. | N.A. | ^[20]^ |
| NiFe LDH //Co_0.07_Ce_0.93_O_2−δ_ | N.A. | N.A. | 2.05 | ^[21]^ |
| Ru-NiMoO(P)_4_ | 1.78 | 2.43 | N.A. | ^[10]^ |
| Ni(OH)_2_  /L-LFP//Pt/C | 1.799 | N.A. | N.A. | ^[28]^ |
| S-(Ni,Fe)OOH //NiMoN | 1.661 | 1.837 | 1.951 | ^[2]^ |
| NiFeS/NF | 1.67 | 1.85 | N.A. | ^[23]^ |
| P-Fe_2_O_3_-CoP | 1.82 | N.A. | N.A. | ^[29]^ |
| Cr-Co_x_P | 1.85 | N.A. | N.A. | ^[30]^ |
| 1D-Cu@ Co-CoO/Rh | 1.95 | N.A. | N.A. | ^[31]^ |
| Ru-CoO_x_/NF | 1.86 | N.A. | 2.62 | ^[32]^ |
| Mo-CoP_x_/NF | 2.16 | N.A. | N.A. | ^[33]^ |
| Ru SAs-MoO_3_-x/NF | 1.759 | N.A. | N.A. | ^[34]^ |
| caMo-NiFePO/NMF | 1.70 | 1.88 | N.A. | ^[35]^ |
| Fe-doped Ni_2_P | 1.74 | 2.32 | N.A. | ^[36]^ |
| Fe_3_Se_4_/NiSe_2_@MXenee | 1.56 | 1.88 | N.A. | ^[37]^ |
| Ru-Ni_2_P/Fe_2_P | 1.727 | 1.912 | N.A. | ^[38]^ |
| Mo-NiP@NF | 1.63 | 1.86 | 1.97 | ^[39]^ |
| Fe-Ni_2_P_v_ | N.A. | 1.79 | 1.86 | ^[40]^ |
| Ag/NiFeRu LDH ‖NiMo/MoO_2_ | N.A. | 1.64 | 1.73 | ^[41]^ |

References

[1] Y. Sun, Z. Xue, Q. Liu, Y. Jia, Y. Li, K. Liu, Y. Lin, M. Liu, G. Li, C. Y. Su, *Nat Commun* **2021**, 12, 1369.

[2] L. Yu, L. Wu, B. McElhenny, S. Song, D. Luo, F. Zhang, Y. Yu, S. Chen, Z. Ren, *Energy Environ. Sci.* **2020**, 13, 3439.

[3] G. Kresse, J. J. C. m. s. Furthmüller, *J. Comp. Mater. Sci.* **1996**, 6, 15.

[4] P. E. Blöchl, *Phys. Rev. B* **1994**, 50, 17953.

[5] J. P. Perdew, J. A. Chevary, S. H. Vosko, K. A. Jackson, M. R. Pederson, D. J. Singh, C. Fiolhais, *Phys. Rev. B* **1992**, 46, 6671.

[6] J. K. Nørskov, J. Rossmeisl, A. Logadottir, L. Lindqvist, J. R. Kitchin, T. Bligaard, H. Jónsson, *J. Phys. Chem. B* **2004**, 108, 17886.

[7] J. K. Nørskov, T. Bligaard, A. Logadottir, J. Kitchin, J. G. Chen, S. Pandelov, U. Stimming, *J. Electrochem. Soc.* **2005**, 152, J23.

[8] D. Pan, B. Yu, J. Tressel, S. Yu, P. Saravanan, N. Sangoram, A. Ornelas‐Perez, F. Bridges, S. Chen, *Adv. Sci.* **2025**, 12, 2414534.

[9] L. Yang, R. Liu, L. Jiao, *Adv. Funct. Mater.* **2020**, 30, 1909618.

[10] S. Wu, D. Chen, S. Li, Y. Zeng, T. Wang, J. Zhang, J. Yu, S. Mu, H. Tang, *Adv. Sci.* **2023**, 10, 2304179.

[11] R. Liu, M. Sun, X. Liu, Z. Lv, X. Yu, J. Wang, Y. Liu, L. Li, X. Feng, W. Yang, B. Huang, B. Wang, *Angew. Chem. Int. Ed.* **2023**, 62, e202312644.

[12] A. Pei, R. Xie, Y. Zhang, Y. Feng, W. Wang, S. Zhang, Z. Huang, L. Zhu, G. Chai, Z. Yang, Q. Gao, H. Ye, C. Shang, B. H. Chen, Z. Guo, *Energy Environ. Sci.* **2023**, 16, 1035.

[13] M. Xing, X. Guo, W. Yuan, W. Chen, M. Du, L. Cai, V. Nicolosi, Y. Chai, B. Qiu, *J. Mater. Chem. A* **2023**, 11, 22147.

[14] G. Liu, J. Zhang, H. Ren, Y. Tang, H. Sun, *Mater. Chem. Front.* **2023**, 7, 4100.

[15] Y. Zhang, K. E. Arpino, Q. Yang, N. Kikugawa, D. A. Sokolov, C. W. Hicks, J. Liu, C. Felser, G. Li, *Nat. Commun.* **2022**, 13, 7784.

[16] L. Guo, T. Liu, L. Zhang, M. Ma, P. Gao, D. Cao, D. Cheng, *Adv. Energy Mater.* **2025**, 15, 2402558.

[17] Y. Li, J. Guo, R. Wang, Y. Zhao, Q. Wang, J. Li, T. Ling, *ACS Appli. Mater. Interfaces* **2025**, 17, 7784.

[18] M. A. Habib, S. Lin, M. H. Joni, S. A. Dristy, R. Mandavkar, J.-H. Jeong, J. Lee, *J. Energy Chem.* **2025**, 100, 397.

[19] J. Sun, Z. Zhao, Z. Li, Z. Zhang, R. Zhang, X. Meng, *J. Mater. Chem. A* **2023**, 11, 22430.

[20] Y. Lu, Z. Zhao, X. Liu, X. Yu, W. Li, C. Pei, H. S. Park, J. K. Kim, H. Pang, *Adv. Sci.* **2024**, 12, 2413245.

[21] G. Su, Y. Hou, J. Yin, J. Yang, Z. Li, X. Du, X. Zhang, P. Xi, C. H. Yan, *Adv. Sci.* **2025**, 12, 2411845.

[22] P. Wang, P. Wang, T. Wu, X. Sun, Y. Zhang, *Adv. Sci.* **2024**, 11, 2407892.

[23] J. Chen, L. Zhang, J. Li, X. He, Y. Zheng, S. Sun, X. Fang, D. Zheng, Y. Luo, Y. Wang, J. Zhang, L. Xie, Z. Cai, Y. Sun, A. A. Alshehri, Q. Kong, C. Tang, X. Sun, *J. Mater. Chem. A* **2023**, 11, 1116.

[24] L. He, Z. Cai, D. Zheng, L. Ouyang, X. He, J. Chen, Y. Li, X. Guo, Q. Liu, L. Li, W. Chu, S. Zhu, X. Sun, B. Tang, *J. Mater. Chem. A* **2024**, 12, 2680.

[25] L. Yu, Q. Zhu, S. Song, B. McElhenny, D. Wang, C. Wu, Z. Qin, J. Bao, Y. Yu, S. Chen, Z. Ren, *Nat. Commun.* **2019**, 10, 5106.

[26] C. Fu, W. Hao, J. Fan, Q. Zhang, Y. Guo, J. Fan, Z. Chen, G. Li, *Small* **2022**, 19, 2205689.

[27] X. Liu, X. Wang, K. Li, J. Tang, J. Zhu, J. Chi, J. Lai, L. Wang, *Angew. Chem. Int. Ed.* **2023**, 63, e202316319.

[28] Z. Li, M. Li, Y. Chen, X. Ye, M. Liu, L. Y. S. Lee, *Angew. Chem. Int. Ed.* **2024**, 63, e202410396.

[29] Z. Cui, Z. Yan, J. Yin, W. Wang, M.-E. Yue, Z. Li, *J. Colloid. Interf. Sci.* **2023**, 652, 1117.

[30] Y. Song, M. Sun, S. Zhang, X. Zhang, P. Yi, J. Liu, B. Huang, M. Huang, L. Zhang, *Adv. Funct. Mater.* **2023**, 33, 2214081.

[31] P. K. L. Tran, D. T. Tran, D. Malhotra, S. Prabhakaran, D. H. Kim, N. H. Kim, J. H. Lee, *Small* **2021**, 17, 2103826.

[32] D. Wu, D. Chen, J. Zhu, S. Mu, *Small* **2021**, 17, 2102777.

[33] Y. Yu, J. Li, J. Luo, Z. Kang, C. Jia, Z. Liu, W. Huang, Q. Chen, P. Deng, Y. Shen, *Mater. Today Nano* **2022**, 18, 100216.

[34] D. Feng, P. Wang, R. Qin, W. Shi, L. Gong, J. Zhu, Q. Ma, L. Chen, J. Yu, S. Liu, S. Mu, *Adv. Sci.* **2023**, 10, e2300342.

[35] P. Tian, W. Zong, J. Xiong, W. Liu, J. Liu, Y. Dai, J. Zhu, S. Huang, S. Song, K. Chu, G. He, N. Han, *Adv. Funct. Mater.* **2025**, DOI: 10.1002/adfm.202504862.

[36] A. Muthurasu, T. H. Ko, T. W. Kim, K. Chhetri, H. Y. Kim, *Adv. Funct. Mater.* **2024**, 34, 2404254.

[37] F. O. Boakye, F. u. Zaman, H. Zhang, A. Saeed, F. T. Dajan, S. Iqbal, K. Harrath, *Adv. Funct. Mater.* **2025**, 35, 2424718

[38] X. Li, T. Wu, N. Li, S. Zhang, W. Chang, J. Chi, X. Liu, L. Wang, *Adv. Funct. Mater.* **2024**, 34, 2400734.

[39] W. Hao, X. Ma, L. Wang, Y. Guo, Q. Bi, J. Fan, H. Li, G. Li, *Adv. Energy Mater.* **2025**, 15, 2403009.

[40] X. Liu, Q. Yu, X. Qu, X. Wang, J. Chi, L. Wang, *Adv. Mater.* **2024**, 36, 2307395.

[41] H. Chen, R. T. Gao, H. Chen, Y. Yang, L. Wu, L. Wang, *Adv. Funct. Mater.* **2024**, 34, 2315674.
